# Identification of environment types and adaptation zones with self-organizing maps; applications to sunflower multi-environment data in Europe

**DOI:** 10.1007/s00122-022-04098-9

**Published:** 2022-05-07

**Authors:** Daniela Bustos-Korts, Martin P. Boer, Jamie Layton, Anke Gehringer, Tom Tang, Ron Wehrens, Charlie Messina, Abelardo J. de la Vega, Fred A. van Eeuwijk

**Affiliations:** 1 Biometris, Wageningen University and Research Centre, Wageningen, The Netherlands; 2 Corteva Agriscience, Ferme Barbara - 265, Route de Boutoli, 82700 Montech, France; 3 Corteva Agriscience, 7300 62nd Avenue, Johnston, IA 50131 USA; 4 Horticultural Sciences Department, University of Florida, 2550 Hull Rd, Gainesville, FL 32611 USA

## Abstract

**Key message:**

We evaluate self-organizing maps (SOM) to identify adaptation zones and visualize multi-environment genotypic responses. We apply SOM to multiple traits and crop growth model output of large-scale European sunflower data.

**Abstract:**

Genotype-by-environment interactions (G × E) complicate the selection of well-adapted varieties. A possible solution is to group trial locations into adaptation zones with G × E occurring mainly between zones. By selecting for good performance inside those zones, response to selection is increased. In this paper, we present a two-step procedure to identify adaptation zones that starts from a self-organizing map (SOM). In the SOM, trials across locations and years are assigned to groups, called units, that are organized on a two-dimensional grid. Units that are further apart contain more distinct trials. In an iterative process of reweighting trial contributions to units, the grid configuration is learnt simultaneously with the trial assignment to units. An aggregation of the units in the SOM by hierarchical clustering then produces environment types, i.e. trials with similar growing conditions. Adaptation zones can subsequently be identified by grouping trial locations with similar distributions of environment types across years. For the construction of SOMs, multiple data types can be combined. We compared environment types and adaptation zones obtained for European sunflower from quantitative traits like yield, oil content, phenology and disease scores with those obtained from environmental indices calculated with the crop growth model Sunflo. We also show how results are affected by input data organization and user-defined weights for genotypes and traits. Adaptation zones for European sunflower as identified by our SOM-based strategy captured substantial genotype-by-location interaction and pointed to trials in Spain, Turkey and South Bulgaria as inducing different genotypic responses.

**Supplementary Information:**

The online version contains supplementary material available at 10.1007/s00122-022-04098-9.

## Background

To produce well-adapted varieties, breeders select candidate genotypes in multi-environment trials that cover a set of locations across various years. These trials are expected to represent the ‘target population of environments’ (TPE), which is the set of likely growing conditions experienced by varieties when grown by farmers in the future (Comstock and Moll 1963; Chapman et al. 2000; Hammer et al. 2019). The TPE can be characterized by a combination of meteorological, soil, and management variables. Genotypes commonly differ in their sensitivity to these variables, leading to genotype-by-environment interactions (G × E) that potentially change the genotypic ranking across environmental gradients. If G × E is large, the genetic gain could be higher when subdividing the TPE and selecting for specific adaptation to geographical and/or management subsets of the TPE (Annicchiarico et al. 2005; de la Vega and Chapman 2006; Atlin et al. 2011). A TPE evaluation for subdivision can be based on advanced breeding material (i.e. candidate varieties) or a reference set of genotypes. A reference set typically contains genotypes which represent the elite germplasm in a breeding program and that discriminate among the target environments (Fox and Rosielle 1982).

To select for specific adaptation, it is crucial to have a reliable characterization of the G × E structure, obtainable from a large sample of locations and years. Such G × E structure can be driven by repeatable (repeating) and non-repeatable elements. Repeatable elements occur when differential genotypic responses and their relationship to environmental drivers of G × E can be estimated reliably. In other words, when the drivers of G × E have been identified and genotypic reaction norms have been estimated and these explain a large proportion of the G × E structure. Genotypic reaction norms can be estimated as a function of continuous or discrete environmental characterizations. An example of continuous environmental characterization is the use of factorial regression or spline-type of models that describe genotypic responses as functions of continuous variables or environmental indices (Millet et al. 2019; Bustos-Korts et al. 2021). Discrete environmental characterizations commonly involve the classification of year-by-location combinations (trials) into environment types, or scenarios of environmental quality that drive adaptation.

The repeatable G × E elements that remain consistent across years for a specific location or management practice, or for which the distribution is known, are referred to as predictable G × E because they can be predicted before planting. Frequently, predictable G × E directly relates to genotype-by-location interactions, G×L (Gauch and Zobel 1997; Annicchiarico 2002). Typical examples of predictable G × E patterns are those driven by soil conditions or photoperiod, or by other geographic characteristics, like latitude, longitude, and altitude.

If predictable G × E, often associated with G×L, describes a large proportion of G × E variance, genetic gain benefits from classifying locations into adaptation zones (also called regions or mega- environments). An adaptation zone is defined as a set of geographical locations with fairly homogeneous growing conditions that cause similar genotypes to perform best across years (Gauch and Zobel 1997). Thus, adaptation zones define predictable G × E, and breeders may select for specific adaptation to each of them. A rationale behind this strategy can be based on comparing direct selection in the adaptation zones, i.e. subdividing the full set of trials, with indirect selection across the TPE, i.e. the undivided full set of trials (Atlin et al. 2000, 2011; de la Vega and Chapman 2006, 2010).

In contrast, G × E is automatically non-predictable when the G × E is non-repeatable and no environmental drivers of G × E can be identified. Alternatively, for repeatable G × E, the environmental drivers of G × E may be known, but their distribution across years can be unknown. In that situation, it is hard to predict which genotypes will perform best at which location (Annicchiarico et al. 2006), and trying to classify locations into adaptation zones or mega-environments is unlikely to pay off in terms of genetic gain.

There are several methods to group and classify trials. When only phenotypic information is available, a popular two-step approach is to first estimate scores for environments (trials) within an application of the Additive-Main-Effects-and-Multiplicative-Interactions model (AMMI, Gauch and Zobel, 1997) or the Genotype-Genotype-by-Environment model (Cooper and DeLacy 1994; Yan and Kang 2002). In a second step, the environmental scores are used for a grouping or clustering of environments into environmental groups, scenarios, types, mega-environments, etc. A comparable strategy has been proposed using environmental scores obtained from estimates for the variance-covariance matrix between trials in a mixed model context (e.g. Cullis et al., 2010). When environmental variables or indices are available (e.g. meteorological or soil variables, or indices calculated with a crop growth model), environments can directly be clustered on such environmental characterizations (Chapman et al. 2000; Chenu et al. 2013; Millet et al. 2016; Bustos-Korts et al. 2019). As environment quality is often a function of many environmental variables, it is advised to integrate environmental information into environmental indices that supposedly better represent environmental signals that influence crop adaptation. Crop growth models are an increasingly popular and useful tool to construct such indices that can be used as input for environment classification (Millet et al. 2019; Rincent et al. 2019; Casadebaig et al. 2022; McCormick et al. 2021; Robert et al. 2020; Costa-Neto et al. 2021). Furthermore, the use of crop growth models provides interesting opportunities to predict genotypic responses to environmental types that might become more frequent in the future due to climate change (Röotter et al. 2013; Dettori et al. 2017; Peng et al. 2020).

A dimension reduction technique that seems particularly suitable for grouping environments into biologically meaningful groups is provided by self-organizing maps (SOMs, Kohonen, 1982). SOMs map objects, environments, or trials in our case, corresponding to multivariate data vectors to a set of points located on a flexible grid or lattice within a two-dimensional coordinate system. The grid points coincide with groups of objects, called units, that themselves represent a first level of clustering. However, it is often useful to achieve a further aggregation of objects by clustering the units into regions or zones within the lattice coordinate system. The joint use of SOM assignment and clustering results in a two-dimensionally smooth k-Means-like clustering that is extremely powerful for visualisation of environmental groupings as well as for showing how genotypes respond to environmental conditions. SOMs have been applied in several fields including biology (van Treuren et al. 2020), characterization of hybrid stability (Clovis et al. 2020) and the analysis of high-throughput phenotyping data (Chen et al. 2014; Singh et al. 2016). However, to our knowledge, they haven’t been used in the context of G × E analysis and environment classification.

In this paper, we aimed to explore the potential of SOMs to classify trials into environment types (focusing on repeatable G × E) and to use those environment types to classify locations into adaptation zones. We identified adaptation zones by clustering locations based on the distribution of environment types per location. That way, locations that share the most frequent environment type across years were classified as belonging to the same adaptation zone. We evaluated the SOM approach for environment classification by quantifying the response to selection across adaptation zones and by quantifying the quality of target trait predictions when classifying trials into environment types or adaptation zones. As input to the SOMs, we used a large-scale sunflower trial network grown in European environments. This trial network was characterized by phenotypic data (grain yield, oil concentration, and flowering time). We also compared the classification results obtained with yield only with those obtained by adding environmental indices calculated with the crop growth model Sunflo (Casadebaig et al. 2011) for specific growth stages, and disease scores.

## Methods

**Fig. 1:**
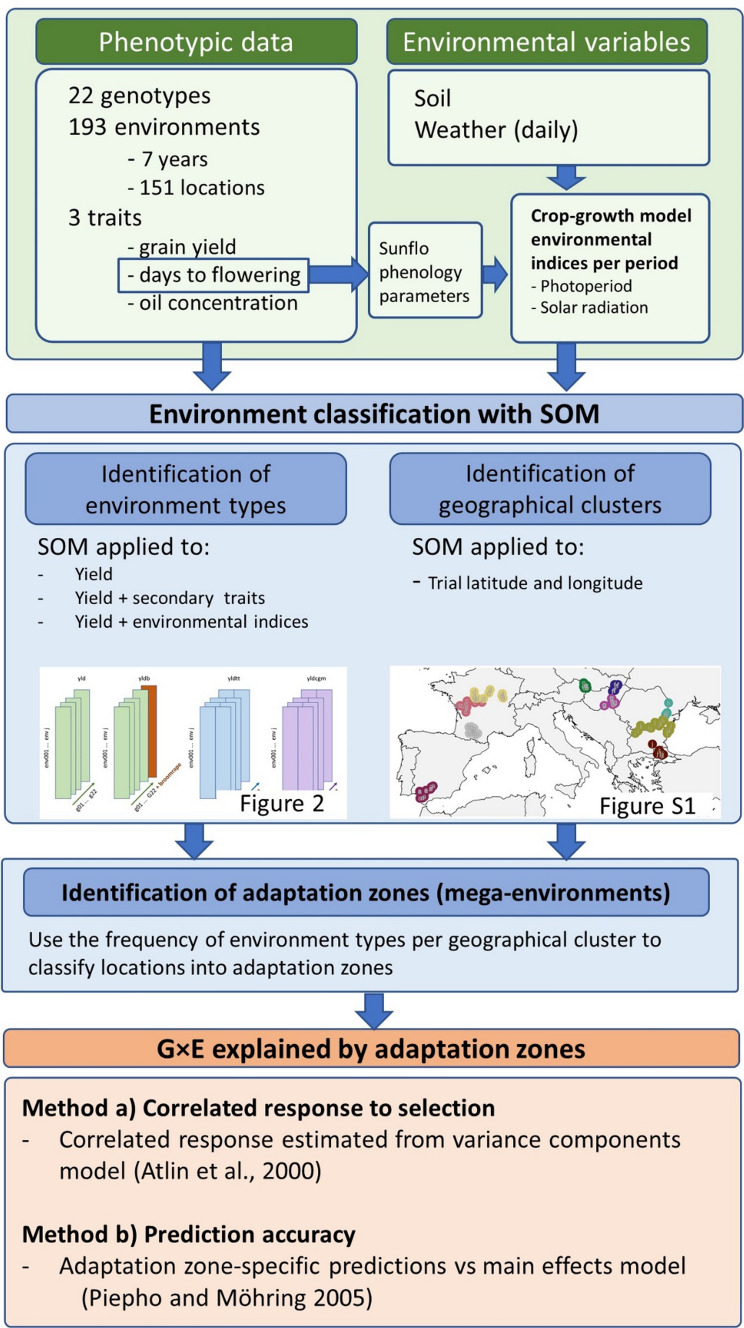
Schematic representation of the modelling steps undertaken to identify adaptation zones and to quantify their contribution to G × E

An overview of our strategy to identifying environment types and adaptation zones for the European sunflower data is given in Fig. 1. In detail, descriptions of our strategy follow below.

### Data

#### Phenotypic data

Phenotypic data consisted of grain yield at 11% moisture, days to flowering (flowering considered to occur when 50% of the plants reached R5, Schneiter and Miller 1981), and grain oil concentration for 22 sunflower hybrids (reference genotypes) grown across 273 locations between 2012 and 2018 (348 trials or environments). Broomrape (*Orobanche cumana* Wallr.) incidence scores were recorded per trial, with two levels; ‘high’ for trials with broomrape pressure, and ‘low’ for trials without broomrape incidence. The hybrids were chosen to represent material adapted to European conditions, ranging from Spain to Ukraine and Russia (Fig. 1 and Figure S1). Genotypes included the phenology range that is relevant to this European TPE. Not all traits were observed in all environments; yield was observed in 348 environments and oil concentration was observed in 193 environments, whereas days to flowering was observed in 138 environments. Most of the analyses were applied to the set of 193 trials that contained yield and oil concentration phenotypes. Some of the methods were evaluated for the full set of 348 trials containing only grain yield. To assess the benefit of additional traits, we considered three trait subsets; only yield, yield plus secondary trait and yield plus crop growth model variables that describe environmental quality (Table 1).

**Table 1:** Input data sets used in sunflower trial classification. The data sets differed in the span of the geographical region and in the traits and covariables that were considered

Data	Traits
Yield (yld)	Grain yield
Yield + Traits (tr)	Grain yield, thermal time to flowering (R1), oil concentration and broomrape incidence score
Yield + CGM (cgm)	Grain yield, crop growth model-calculated covariables

Experiments were laid out as a row-column (spatial) design with one or two replicates, plus additional (diagonal) checks to account for spatial variability. Experimental units consisted of 4-row plots of 6 m long that were machine harvested after maturity. Cultural practices corresponded to those typical of the sampled growing areas.

#### Estimation of adjusted means per trial for the phenotypic data

To separate genotypic effects as good as possible from within-trial noise, adjusted means for genotypes were estimated per trial with the following mixed model:

1 $$\underline {y}_{irc} = \mu + \underline {R}_{r} + \underline {C}_{c} + G_{i} + \underline {\epsilon }_{irc}$$

where $\underline {y}_{irc}$ is the phenotype of genotype *i* observed in row *r* and column *c* of the trial, $\mu$ is an intercept, $\underline {R}_{r}$ is a random effect for row *r*, $\underline {C}_{c}$ a random effect for column *c*, $G_{i}$ is the fixed effect of genotype *i*, and $\underline {\epsilon }_{irc}$ is a residual. Row and column effects are assumed to follow normal distributions with variances $\sigma_{r}^{2}$ and $\sigma_{c}^{2}$, respectively. The residual $\underline {\epsilon }_{irc}$ was modelled with a first order autoregressive structure along rows and columns (Gilmour et al. 1997). The adjusted genotypic means, called best linear unbiased estimators or BLUEs, were carried forward to subsequent G × E analyses, with the reciprocals of the corresponding squared standard errors serving as weights.

#### Environment data

Environment data and plant density were used as input to the Sunflo model. Climatic data (minimum and maximum temperature, solar radiation, rainfall, vapour pressure deficit) were extracted from the weather database provided by the IBM Weather Company (IBM 2021). Soil information (soil depth and other variables related to water retention capacity) was extracted from the Harmonized World Soil Database (FAO/IIASA/ISRIC/ISSCAS/JRC 2012). Plant density was recorded at the trial level.

### Using self-organizing maps to classify trials into environment types

A self-organizing map is an unsupervised machine learning method to identify patterns in data by mapping high-dimensional data to a low and typically two-dimensional set of coordinates or points organized in a grid or lattice so that the topological structure of the data in the high-dimensional space is preserved in the low dimensional space. Grid points correspond to typical combinations of variable or feature values that are called ‘prototypes’ or ‘codebook vectors’. Individual objects in the data are assigned to the closest prototype and form groups or ‘units’, where closest is defined with respect to a distance metric like the Euclidean distance. As a result, the position of grid points, and corresponding prototypes and units, in a SOM is determined by the distances to neighbouring points, in contrast to techniques like principal components analysis (PCA), where the direction of the largest principal components is determined by the most outlying points. Essential differences with traditional dimension reduction techniques in plant breeding, like PCA and reduced rank regression (van Eeuwijk 1992; Graffelman and Van Eeuwijk 2005), are that SOMs can deal with non-linearities in the high-dimensional data space and tend to distribute dense parts of the data space over several (neighbouring) units, making it easier to assess the presence of substructures (Wehrens 2020). The choice of the grid dimensions, and with that the resolution of the grid relative to the objects that require assignment to prototypes and units, is a ‘parameter’ under control of the user. The grid size is a compromise between within-unit heterogeneity and unit size, i.e. a too large grid will have a too high resolution and will lead to units with no or few objects, while a too small grid will have too low resolution and strong unit heterogeneity for some units. We used a grid size of 5 by 5 because it provided sufficient resolution without creating empty units.

#### Data representation and preparation for SOM analysis

To train a SOM, data need to be arranged in one or more vectors or matrices, with the objects that will be classified labelling the rows and with variables in the columns. Each of the data vectors or matrices that are used is called a ‘layer’ and contains a different piece of information about the trials. In each layer, variables can correspond to one or more continuous random variables (in our sunflower data, the genotypic BLUEs for grain yield, other traits, or environmental indices), or discrete variables (in our sunflower data, the indicator for broomrape incidence within each trial). We can think of our trial data as follows: trials define the objects we want to classify, the data on which the classification will be based are organized in layers, and each layer contains at least one trait or variable, although often a layer contains multiple traits or variables. Therefore, the dimensions of layers are always the number of objects (trials) × the number of traits/variables. Heterogeneous traits/variables within a layer require normalization or scaling. For each layer, a unique distance measure can be chosen to define distances between objects. SOM algorithms can deal with missing values, but we preferred to impute missing yield and oil concentration values beforehand by fitting an additive model with fixed genotype and environment effects and using those fitted values to fill in the missing genotype-environment combinations in the data. These imputed data were used exclusively for the SOM analysis part. Subsequent analyses based on mixed models used the original non-imputed data.

As we wanted to classify trials into environment types that explain G × E and preserve genotypic ranks within environment types, we scaled continuous traits and variables per trial to have zero mean and unit standard deviation. Note that this is the same kind of scaling that is used to explore correlations between variables in a heterogeneous set of variables by PCA. Effectively, we concentrated in the SOM analysis on the genotypic correlations between trials. Without this scaling step, trials would be primarily classified on the basis of their means, i.e. the environmental main effects, and less by their ability to discriminate between genotypes within trials.

**Fig. 2:**
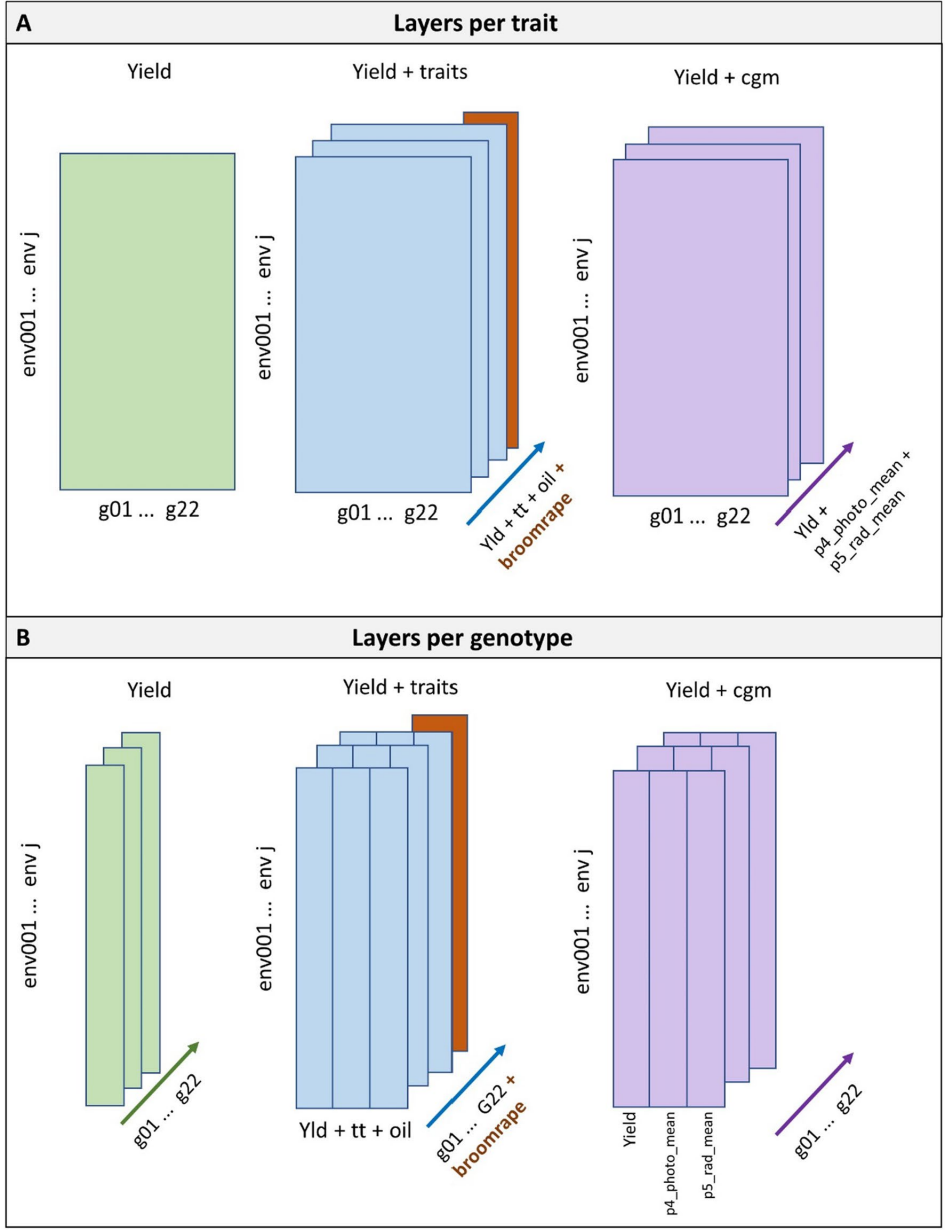
Schematic view of input data organization for different applications of self-organizing maps (SOMs). **A** Layers arranged as one layer per trait, where each layer is a matrix with the environments in the rows and genotypes in the columns (except for the broomrape incidence scores which have one value per environment only). **B** Layers arranged as one layer per genotype. In each genotypic layer, rows correspond to environments and columns to yield, secondary traits or crop growth model environmental indices. *Yield* corresponds to a SOM applied to yield only; *yield* + *traits* corresponds to a SOM applied to yield plus thermal time to flowering, oil concentration and broomrape scores and *yield* + *cgm* corresponds to a SOM applied to yield, plus the environmental indices calculated with the crop growth model Sunflo: mean photoperiod between flowering and onset of senescence (p4_photo_mean), and mean solar radiation between the onset of senescence and maturity (p5_rad_mean)

In the analysis of our sunflower data, we used two types of input data layer arrangements: (i) a single layer per trait, with the columns within that layer corresponding to genotypes, and (ii) a single layer per genotype, with multiple layers corresponding to different genotypes (Fig. 2). In the arrangement of one layer per trait, each layer consisted of a matrix of dimensions 193 (or 348) environments by 22 genotypes. In the arrangement of one layer per genotype, each layer consisted of a matrix of 193 (or 348) environments by the number of traits considered in that specific analysis. In this latter arrangement, genotype is interpreted as a bio-assay of the environmental conditions following the concept of ‘reference’ genotypes (Fox and Rosielle 1982).

#### Distance measures

The R package ‘kohonen’ provides a flexible implementation for SOM, which allows using a large variety of distance measures (Wehrens and Kruisselbrink 2018). In this paper, we used sum-of-squares distances for layers with quantitative traits and environmental covariables, and Tanimoto distances (equivalent to Jaccard distances, Härdle and Simar 2013) for the layer containing broomrape incidence scores.

#### Layer weights

Distance functions or metrics can be defined for each individual layer included in a SOM analysis. The distances are then combined across layers retaining the assigned distance metrics per layer and allowing for an additional user-defined weight per layer. In the arrangement of one layer per trait, with yield plus other traits (oil concentration, thermal time to flowering and broomrape incidence scores), we assigned a weight of 3 to yield, 2 to oil concentration, 1 to thermal time to flowering and 1 to the broomrape scores. Layer weights were user-defined parameters. In this example, we chose weights that roughly express the assumed biological closeness to the target trait. For the arrangement of one layer per trait, with yield plus the crop growth model indices ‘p4_photo_mean’ and ‘p5_rad_mean’ we assigned a weight of 4 to yield and a weight of 1 to each of the crop growth model indices to assign more importance to the trait of economic interest. In the arrangement of one layer per trait, genotypes are arranged as columns within each of the trait matrices. Therefore, all genotypes receive automatically the same weight.

Following the idea of probe genotypes, genotypes with a known reaction to identified environmental stresses, we wanted to evaluate whether giving more weight to genotypes that respond stronger to the environment would lead to a more pronounced identification of adaptation zones. Therefore, in the arrangement of one layer per genotype, we weighted genotypic layers by the genotypic contribution to G × E. (The order of the layers does not influence the results; therefore, we just ordered them alphabetically.) The squared lengths of the AMMI genotypic vectors will give the 2-dimensional approximation to the sums of squares for interaction for individual genotypes, provided the biplot is made with genotype scaling, i.e. the genotypic scores are calculated as singular vector multiplied by the singular value. However, to stay away from the graphics in the biplot, one can easily calculate the sums of squares for interaction for each genotype in an AMMI-2. This was done and the sums of squares were used as weights in the SOM. This led to tenfold differences in genotype weights (the genotype with the smallest weight was gen06 and the one with the largest weight was gen14, Figure S2). Broomrape scores were considered as an additional layer. Broomrape scores were given a weight equal to the average genotype weight for yield (i.e. 4.46).

#### Training a SOM

The goal of training a SOM is to obtain a low dimensional grid of points with corresponding prototypes and units that approximates the distribution of the originally high-dimensional data and that reveals hopefully biologically interpretable patterns in the data by keeping objects that were close together in the high-dimensional space also close together in the low-dimensional grid. The initial prototypes were taken randomly and are adjusted during an iterative process in which all objects are repeatedly presented to the prototypes. In each iteration, the most similar unit, called the ‘winning unit’, is rotated slightly towards the presented object, which is a process reminiscent of K-means clustering. What distinguishes SOMs is that not only the winning unit but also its immediate neighbourhood is rotated, albeit to a lesser degree. During training, the size of this neighbourhood gradually decreases until, in the end, only K-means-like updates are made (Kohonen 1982).

#### Clustering step

A final assignment of trials to environment types was done by a further clustering of the SOM prototypes. Without this step, there would be as many environment types as units in the map. We chose for a hierarchical clustering of the prototypes, using the function ‘hclust’ in R. We considered dendrogram cuttings leading to six environment types. A smaller number of environment types produced too similar environment type frequencies per geographical cluster, leading to a too low resolution for identifying adaptation zones.

### Identification of adaptation zones from environment types

Environment types resulting from the SOMs and subsequent clustering of prototypes focus on the classification of trials driving repeatable G × E. The next step was to use the environment types to identify adaptation zones by grouping location clusters (Figure S1) with a similar distribution of environment types across years. These adaptation zones captured predictable G × E.

#### Clustering locations into geographical groups

As a preliminary first step to identify adaptation zones from environment types, we created geographical units containing nearby locations by clustering trials on latitude and longitude (Figure S1). Here, we assumed that geographically close trials provide a sample of environmentally similar conditions that will belong to the same adaptation zone. For convenience, we used SOMs for this classification, but many alternative methods would have produced similar geographical groupings of trials. We arranged trial latitude and longitude information as separate layers and constructed a SOM with the ‘kohonen’ package in R (Wehrens and Kruisselbrink, 2018). This SOM procedure led to 10 geographical clusters when considering the 193 trials with yield and oil concentration, and to 17 geographical clusters when considering the 348 trials with yield only (Figure S1).

#### Identifying adaptation zones by clustering geographical groups with respect to the frequency of environment types

The relative frequencies of environment types within geographical clusters of trials as identified by SOMs on phenotypic traits and environmental variables and indices changed among those geographical clusters. These relative frequencies of environment types defined Chi-square distances between geographical clusters and formed the input to a hierarchical clustering procedure to identify adaptation zones (Ward's method, Härdle and Simar, 2013), again using the ‘hclust’ function in R (R Core Team 2019). Two geographical clusters belonged to the same adaptation zone if the relative frequencies of their environment types were comparable. As for most of the SOM input variable combinations four adaptation zone clusters were identified, we presented the results with this number of adaptation zones to maintain a certain consistency.

To compare the assignment of trials to adaptation zones between SOMs with different layer arrangements and trait subsets, we calculated so-called balanced accuracies using the R-package caret (Kuhn 2021). When we consider two different SOM applications, say SOM1 and SOM2, then for a particular zone identified by SOM1, say SOM1-a, we can compare with a zone identified by SOM2, say SOM2-b. Call the number of trials assigned to both SOM1-a and SOM2-b ‘A’, the number of trials assigned to SOM1-a and not assigned to SOM2-b ‘B’, the number of trials not assigned to SOM1-a and assigned to SOM2-b ‘C’, and trials assigned to neither of SOM1-a and SOM2-b ‘D’. Balanced accuracy is the average of A/(A + C) and D/(B + D).

### G × E characterization

#### Quantifying repeatable and non-repeatable G × E

To quantify the relative size of predictable vs. unpredictable G × E, we fitted the following mixed model to the BLUEs that followed from trial analyses using the mixed model 1 described earlier, where we carried forward weights for the estimation of effects and variance components in the form of the reciprocal of the squared standard errors of the per trial genotypic BLUEs (Möhring and Piepho 2009; Welham et al. 2010). The model was fitted in ASReml-R (Butler et al. 2019):

2 $$\underline {y}_{ilm} = \mu_{lm} + \underline {G}_{i} + \underline{GL}_{il} + \underline{GY}_{im} + \underline{GLY}_{ilm} + \underline {\epsilon }_{ilm}$$

In model 2, $\underline {y}_{ilm}$ is the yield of the genotype *i* in location *l* and year *m*. There is a trial specific fixed intercept term, $\mu_{lm}$. G × E is decomposed into normally distributed zero mean genotype-by-location interaction ($\underline{GL}_{il}$), genotype-by-year interaction ($\underline{GY}_{im}$), and genotype-by-location-by-year interaction ($\underline{GLY}_{ilm}$), with variance components $\sigma_{gl}^{2}$, $\sigma_{gy}^{2}$, $\sigma_{gly}^{2}$, respectively. $\underline {\epsilon }_{ilm}$ is the error variance (scaled to 1) that was separated from $\sigma_{gly}^{2}$ because we used a weighted analysis (Möhring and Piepho 2009; Welham et al. 2010).

#### Selection of environmental covariables for inclusion in SOMs

We used predicted thermal time to flowering to define phenology parameters that were genotype-specific in the Sunflo crop growth model. After running the Sunflo crop growth model for each genotype-environment combination, we used the Sunflo phenology predictions and stage definitions to describe five developmental periods: (i) between sowing and emergence (p1), (ii) between emergence and floral initiation (p2), (iii) between floral initiation and flowering (p3), (iv) between flowering and onset of senescence (p4), and (v) between the onset of senescence and maturity (p5). Environmental indices were calculated for each genotype-environment combination within each of these developmental periods.

The number of calculated covariables was large (107 covariables, Table S2). For that reason, we used a random factorial regression model together with some prior biological insights about which covariable out of a set of highly correlated covariables will be most likely the causal one to pre-select a smaller number of covariables to be included in the environment classification task with SOMs. We used the following model:

3 $$\underline {y}_{ij} = \mu_{j} + \underline {G}_{i} + \underline {\beta }_{i} z_{ij} + \underline {\epsilon }_{ij}$$

In model 3, $\underline {y}_{ij}$ is the yield of genotype *i* in environment *j*, $\mu_{j}$ is a fixed intercept for environment *j*, $\underline {G}_{i}$ is a random genotypic intercept, $\underline {\beta }_{i}$ is a random slope of genotype *i* with respect to the scaled genotype-specific environmental covariable $z_{ij}$, and $\underline {\epsilon }_{ij}$ is a residual that contains G × E not explained by the covariable, plus within-trial error (no weights were used in model 3). All covariables were scanned one at a time. Among the significant covariables by a standard likelihood ratio test on the variance component, we stored the five covariables that led to the smallest error variance. We then used physiological knowledge (e.g. Villalobos and Ritchie 1992) to select one variable to be retained in the model, out of the five candidates. After inclusion of the selected covariable, the whole process was repeated until no further covariables could be identified anymore that decreased the residual variance.

#### Evaluation of the adaptation zones

To evaluate the suitability of selecting for specific adaptation to identified adaptation zones, we compared the accuracies of prediction models with and without adaptation zones. When identified adaptation zones lead to differential genotype adaptation, prediction accuracies of models with adaptation zones will exceed those of models without adaptation zones.

##### Variance–covariance modelling at the level of adaptation zones

As a reference, we predicted genotypic performance at each trial using the following two-way mixed model without adaptation zones:

4 $$\underline {y}_{ij} = \mu_{j} + \underline {G}_{i} + \underline{GE}_{ij} + \underline {\epsilon }_{ij}$$

In model (4), $\underline {y}_{ij}$ are the BLUEs for genotype *i* in environment *j*, estimated using model (1), $\mu_{j}$ is an environment-specific intercept, $\underline {G}_{i}$ is a zero mean normally distributed effect for genotype *i* with variance $\sigma_{g}^{2}$, $\underline{GE}_{ij}$ is a zero mean normally distributed genotype-by-environment interaction with variance $\sigma_{ge}^{2}$. $\underline {\epsilon }_{ij}$ is the error variance (scaled to 1) that was separated from $\sigma_{ge}^{2}$ because we used a weighted analysis (Möhring and Piepho 2009; Welham et al. 2010).

Model (4) can be converted into a model with adaptation zones in the following way, where the G × E is partitioned into a part due to adaptation zones and residual genotype by trial within adaptation zones.

5 $$\underline {y}_{ij\left( z \right)} = \mu_{j} + \underline{GZ}_{iz} + \underline{GE\left( Z \right)}_{ij\left( z \right)} + \underline {\epsilon }_{ij\left( z \right)}$$

In model (5), $\underline{GZ}_{iz}$ is a normally distributed genotype-by-adaptation zone interaction with zero mean and zone specific variances, $\sigma_{{gz}}^{2}$ and covariances $\sigma_{zz^{\prime}}$, allowing the borrowing of information between adaptation zones (Piepho and Möhring 2005). The terms $\underline{GE \left( Z \right)}_{ij\left( z \right)}$ and $\underline {\epsilon }_{ij\left( z \right)}$ are zero mean normally distributed with variances $\sigma_{ge}^{2}$ and $\sigma_{\epsilon }^{2}$, respectively. As before, weights were equal to reciprocals of squared standard errors. We inspected the predictions of model (5) in an AMMI biplot (Gauch and Zobel 1997).

##### Cross-validation

When comparing models with and without adaptation zones, we assume that the trials included in multi-environment testing are a sample of the TPE. Hence, if adaptation zones were to have an impact on crop adaptation in the long term, they should also explain a portion of the G × E in the sample of the TPE represented by the trials. To investigate this hypothesis, we constructed a leave-one-year-out cross-validation scheme in which a subset of six out of the full set of seven years was assumed to represent a training set. For each subset of six years, we generated predictions from models 4 and 5 and calculated the prediction accuracies for the hold-out trials. Predictions obtained from model 4 were for the genotype main effect, whereas predictions obtained from model 5 were specific to each adaptation zone or mega-environment. Accuracy was calculated for each trial as the correlation between predicted and observed yield.

##### Correlated response to selection

We also quantified the utility of distinguishing adaptation zones via the approach proposed by Atlin et al. (2000), in which responses to selection for divided (with adaptation zones) and undivided (without adaptation zones) TPE are compared. The ingredients for that comparison follow from the fit of the following mixed model for the phenotypic response of genotype *i* at location *l* that is part of zone *z* in year *m*:

6 $$\underline {y}_{ilmz} = \mu_{lm} + \underline {G}_{i} + \underline{GZ}_{iz} + \underline{GL\left( Z \right)}_{il\left( z \right)} + \underline{GY}_{im} + \underline{GZY}_{izm} + \underline{GL\left( Z \right)Y}_{il\left( z \right)m} + \underline {\epsilon }_{ilmz}$$

In model 6, all terms except the trial intercept $\mu_{lm}$ are zero mean normally distributed terms with a unique variance, with $\underline {G}_{i}$ the genotypic main effect, $\underline{GZ}_{iz}$ the genotype-by-adaptation-zone interaction, $\underline{GL\left( Z \right)}_{il\left( z \right)}$ the genotype-by-location-within-adaptation-zone interaction, $\underline{GY}_{im}$ the genotype-by-year interaction, $\underline{GZY}_{izm}$ the genotype-by-adaptation-zone-by-year interaction, $\underline{GL\left( Z \right)Y}_{il\left( z \right)m}$ the genotype-by-location-within-adaptation zone-by-year interaction. The corresponding variances are $\sigma_{g}^{2}$, $\sigma_{gz}^{2}$, $\sigma_{gl\left( z \right)}^{2}$, $\sigma_{gy}^{2}$, $\sigma_{gzy}^{2}$ and $\sigma_{gl\left( z \right)y}^{2}$. $\underline {\epsilon }_{ilmz}$ is the error variance (scaled to 1) that was separated from $\sigma_{gl\left( z \right)y}^{2}$ because we used a weighted analysis (Möhring and Piepho 2009; Welham et al. 2010).

The comparison of the direct response to selection, *DR*, for selecting in a divided TPE, i.e. selection within regions, with the correlated response for selection in an undivided TPE, *CR*, was calculated as in Eq. 7, following (Falconer and Mackay 1996). Ratios of *CR/DR* smaller than 1.0 indicate a larger response when selecting within regions, so subdivision into adaptation zones is worthwhile. Ratios above 1.0 indicate a larger response when selecting across regions, so better not to subdivide into regions.

7 $$\frac{{{\text{CR}}}}{{{\text{DR}}}} = \rho_{g} \sqrt {\frac{{H_{{{\text{Undivided}}}}^{2} }}{{H_{{{\text{Divided}}}}^{2} }}}$$

In Eq. 7, $\rho_{g}$ is the genetic correlation between a phenotypic response as observed in an undivided TPE and a response observed within an adaptation zone. This correlation is expressed as:

8 $$\rho_{g} = \frac{{\sigma_{g}^{2} }}{{\sqrt {\sigma_{g}^{2} \left( {\sigma_{g}^{2} + \sigma_{gz}^{2} } \right)} }}$$

$H_{{{\text{Undivided}}}}^{2}$ and $H_{{{\text{Divided}}}}^{2}$ are the heritabilities, or better, the repeatabilities, of line means in the undivided set of locations and the zones, respectively. Their estimators are functions of variance components, as given in Eqs. 9 and 10, where $nl$,$ny,$ and *nz* are the median number of locations per zone, years, and zones in which genotypes were present.

9 $$H_{{{\text{Undivided}}}}^{2} = \frac{{\sigma_{g}^{2} }}{{\sigma_{g}^{2} + \frac{{\sigma_{gz}^{2} }}{nz} + \frac{{\sigma_{gl\left( z \right)}^{2} }}{nln z} + \frac{{\sigma_{gy}^{2} }}{ny} + \frac{{\sigma_{gzy}^{2} }}{nynz} + \frac{{\sigma_{gl\left( z \right)y}^{2} }}{nl nz ny}}}$$

10 $$H_{{{\text{Divided}}}}^{2} = \frac{{\sigma_{g}^{2} + \sigma_{gz}^{2} }}{{\sigma_{g}^{2} + \sigma_{gz}^{2} + \frac{{\sigma_{gl\left( z \right)}^{2} }}{nl} + \frac{{\sigma_{gy}^{2} }}{ny} + \frac{{\sigma_{gzy}^{2} }}{ny} + \frac{{\sigma_{gl\left( z \right)y}^{2} }}{n ln y}}}$$

## Results

### Variance components for GLY

In this paper we aim at exploring the potential of SOMs to understand the drivers of G × E, classifying trials into environment types (focusing on repeatable G × E), and ultimately, classifying locations into adaptation zones focusing on predictable G × E. We first quantified the relative importance of repeatable vs. non-repeatable G × E comparing magnitudes of variance components as given in Table 2. There was considerable G × E (with G × E being 3.07 times the genotype main effect, Table 2). The relative importance of G × E aligned well with what is expected for the wide range of the genotypes and environments that were included in the sunflower data set. Most G × E variation was unpredictable (73.1% of the G × E corresponded to G × Y + G × L × Y variation), but G × L was large enough to identify adaptation zones.

**Table 2:** Variance components for genotypic main effect and G × E interactions (193 trials)

Source	Component	SE	%G × E
G	14,931	4878	
GY	2167	659	4.74
GL	12,311	2237	26.90
GLY	31,281	2341	68.36

### Characterizing G × E with SOM for yield

This section focuses on the use of SOMs to classify trials into environment types (ETs).

#### Layers per trait

**Fig. 3:**
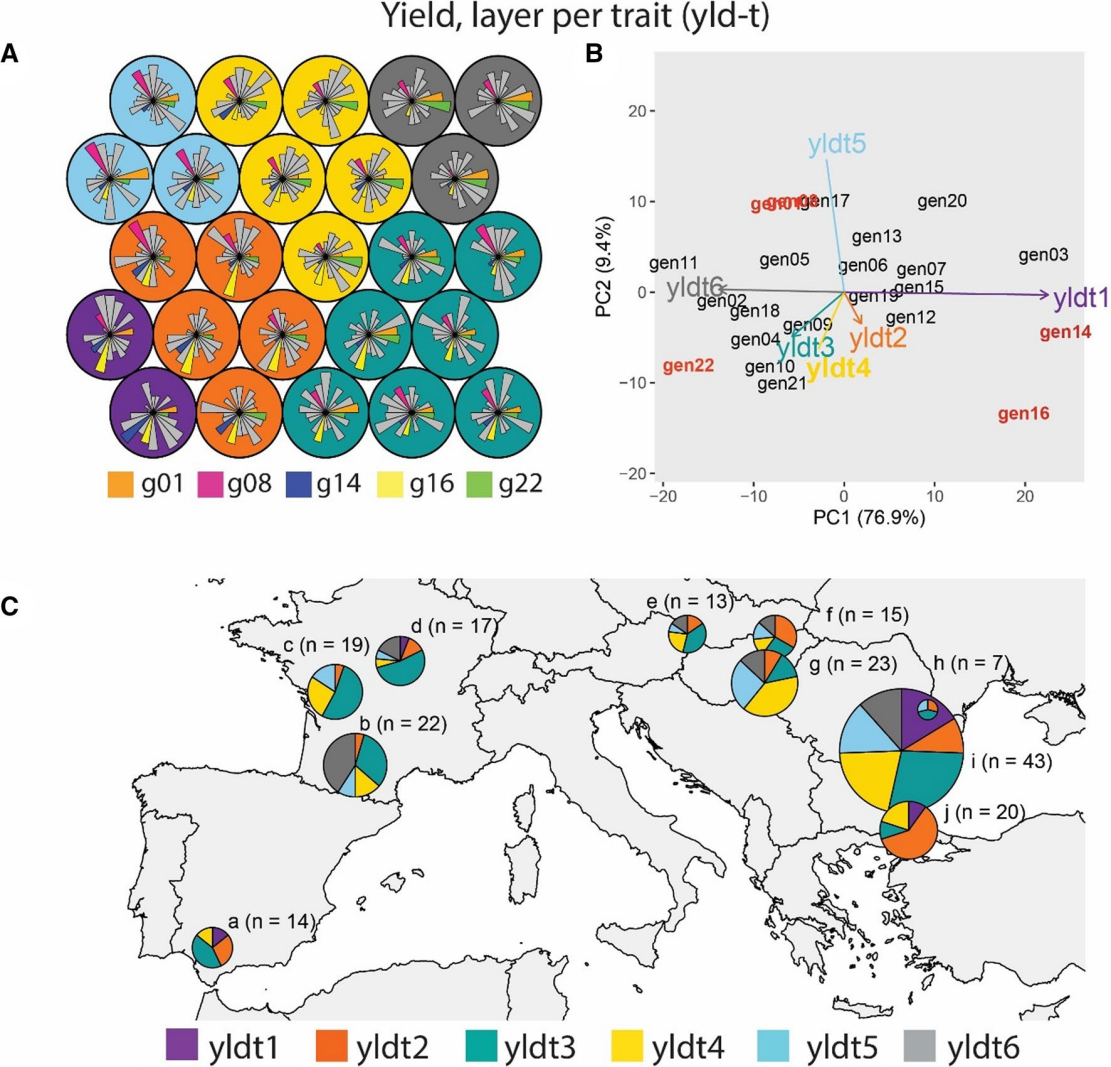
Yield, one layer per trait (yld-t) **A** Prototypes for example genotypes that drive G × E across these environment types. Prototypes (outer circles) are coloured according to their corresponding ET. Genotypes are indicated in sequential order from gen01 to gen22. Radius of each genotype is proportional to the genotype performance in trials belonging to that prototype (a larger diameter means a larger yield, relative to the other genotypes because the yield was standardized within a trial). **B** AMMI biplot for mixed-model predicted yield of genotypes at each environment type, as identified with a self-organizing map, and **C** Map of environment types for yield. Pie sizes are proportional to the number of trials present in that geographical cluster. The ‘n’ next to each pie map indicates the number of trials in that particular geographical cluster

Environment classification using grain yield led to six environment types (ETs, Fig. 3). The AMMI biplot was used for visualization of the genotypic BLUES at each environment type, as predicted by Eq. 4. The SOM can be interpreted in a similar way as an AMMI biplot, with genotypes showing a larger prototype radius at a particular group of environments corresponding to those that show a positive interaction in the AMMI biplot. The SOM-obtained ETs were driven by the contrasting genotypic responses of genotypes like gen01, gen08, gen14, gen16 and gen22 (Fig. 3A). Therefore, we will focus on these five genotypes as an example to make comparisons throughout the paper. In the SOM figures, the codebook vectors have been indicated in colour for the example genotypes.

yldt1 was composed of trials with which gen14 and gen16 had a positive interaction (Fig. 3A, B). yldt1 occurred most commonly in Romania and North of Bulgaria (pie i, Fig. 3C) and less frequently in South of Bulgaria, Turkey and Spain (pies j and a, Fig. 3C). In contrast, yldt6 corresponded to environments with which gen01 and gen22 had a positive interaction. Gen14 and gen16 had a negative interaction with ET6 trials. yldt3 occurred most commonly in the South of France (pie b, Fig. 3C), South of Romania and North of Bulgaria (pie i, Fig. 3C) and less frequently in the other Northern trials (pies c, d, e, f, g and h, Fig. 3C), but it was absent in the Southern trials in Spain, Turkey and South of Bulgaria (pies a and j, Fig. 3C).

#### Layers per genotype

**Fig. 4:**
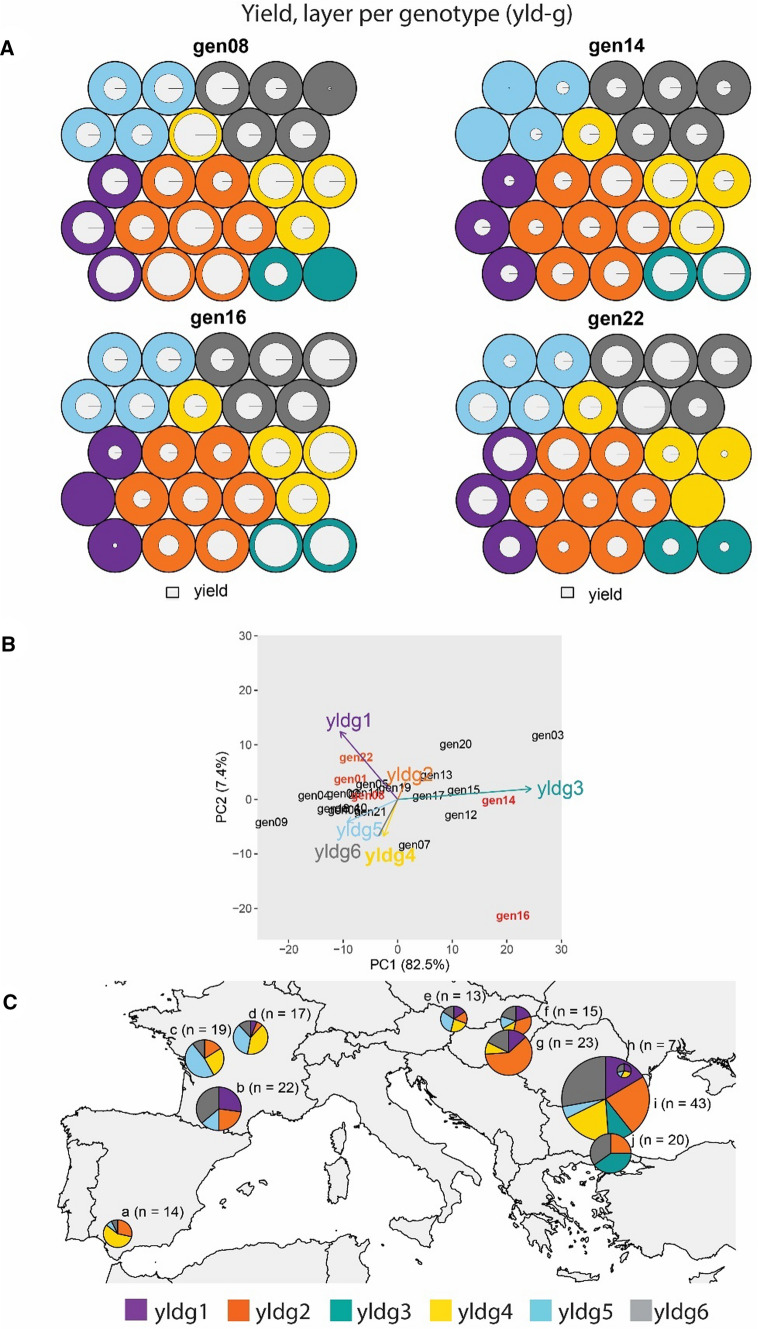
Yield, one layer per genotype (yld-g). **A** Prototypes for example genotypes that drive G × E across these environment types. Prototypes (outer circles) are coloured according to their corresponding ET. Colour codes for ETs are indicated at the bottom of the figure. Inner radius (i.e. white circle) inside each prototype is proportional to the genotype performance in trials belonging to that prototype (a larger radius means a larger yield, relative to the other genotypes because the yield was standardized within a trial). **B** AMMI biplot for mixed-model predicted yield of genotypes at each environment type, as identified with a self-organizing map, and **C** Map of environment ypes for yield. Pie sizes are proportional to the number of trials present in that geographical cluster. The ‘n’ next to each pie map indicates the number of trials in that particular geographical cluster

**Fig. 5:**
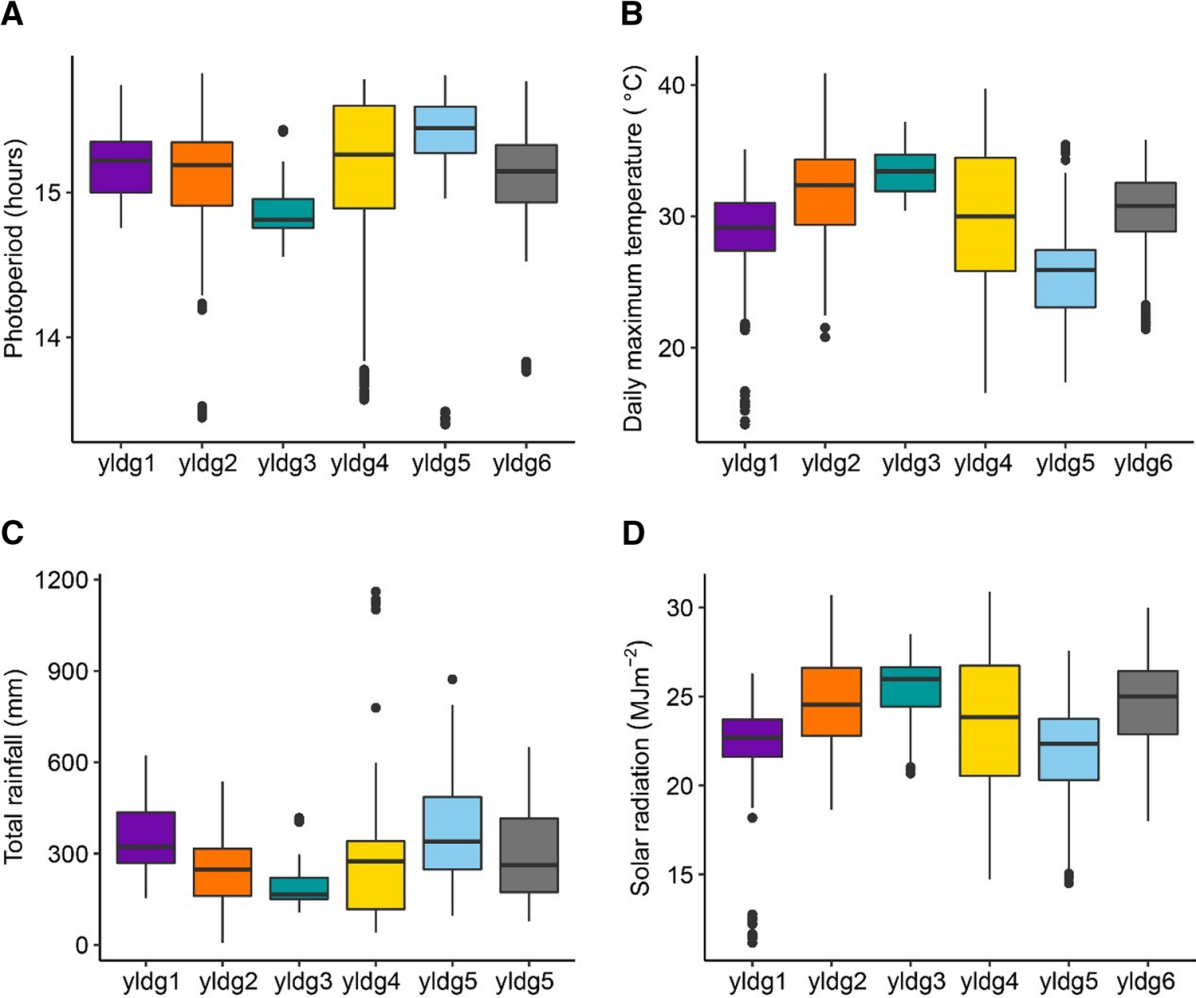
Environmental conditions at each of the environment types identified with the SOM applied to the yield data with one layer per genotype (yldg, same as reported in Fig. 4). **A** Photoperiod between emergence and floral initiation, **B** Daily maximum temperature between the onset of senescence and maturity, **C** Rainfall between emergence and maturity and **D** Mean solar radiation between flowering and onset of senescence

When arranging data as one layer per genotype, we weighted genotypes by their contribution to G × E. Among the six identified ETs (Fig. 4), G × E was driven by the contrast between yldg3 versus yldg1 + yldg5. Gen14 and gen16 showed a strong positive interaction with yldg3, reflected by the large prototype diameter (Fig. 4A) and the large score for AMMI1 (Fig. 4B). yldg3 occurred most often in Romania Bulgaria and Turkey (pies i and j in Fig. 4C). These trials showed shorter photoperiod (mean photoperiod between emergence and floral initiation of 14.9 h), high maximum temperature (mean of 33.6 °C), reduced rainfall (194 mm), higher solar radiation (25.5 MJm^−2^, Fig. 5) and a higher broomrape pressure, compared to the other ETs.

Gen14 and gen16 also showed a negative interaction with yldg1 and yldg5, with a small prototype diameter (Fig. 4A). In contrast, gen22 and gen08 had a positive interaction with environment type yldg1 and yldg5, and a negative interaction with yldg3. Environment type yldg1 occurred most commonly in the South of France, Romania and North of Bulgaria (pies b and i in Fig. 4C), whereas yldg5 had a longer photoperiod (15.4 h), lower mean maximum temperatures (25.9 °C), higher rainfall (371 mm) and lower solar radiation (21.9 MJm^−2^), compared to yldg3, Fig. 5. yldg5 occurred most often in France and Austria (pies c, d and E in Fig. 4C). Spain had a large proportion of trials classified as yldg4, which corresponded to environments with shorter photoperiod and reduced rainfall, compared to the other ETs. Gen22 had a negative interaction with yldg1, whereas gen08, gen14 and gen16 had a positive interaction with yldg1. In the arrangement of layers per genotype, this negative interaction was observed as a smaller prototype diameter, whereas the same interaction was observed as a smaller radius in the SOM applied to one layer per trait (Fig. 3).

### Characterizing G × E with SOM for multiple traits

In this data arrangement, we considered yield, plus the secondary traits thermal time to flowering, oil concentration and scores for broomrape incidence.

#### Layers per trait

When using the layers per trait arrangement, we gave more weight to the target trait yield, and less weight to the other traits. The SOM allows to visualize how each of the traits and genotypes project on the trial groupings.

The arrangement of layers per trait led to six ETs that explained G × E for grain yield (Figure S3A and S3B). The largest contrast was given by trt4 with the other ETs (especially with trt5). trt4 was mainly composed of trials with which gen14 and gen16 had a positive interaction for yield and that had a large broomrape incidence (Figure S3A). For thermal time to flowering, gen08 and gen22 had a positive interaction with trt4 trials, whereas for oil concentration, gen08 and gen16 had a positive interaction with trt4 trials. trt4 trials occurred only in South Romania, Bulgaria and Turkey (pies i and j in Figure S3C). The environment type trt5 corresponded to trials for which gen08, gen16 and gen22 had a positive interaction for yield, whereas gen08 and gen22 had a positive interaction for thermal time to flowering (i.e. got more delayed than other genotypes, a positive G × E effect) and gen16 had a positive interaction for oil concentration.

#### Layers per genotype

**Fig. 6:**
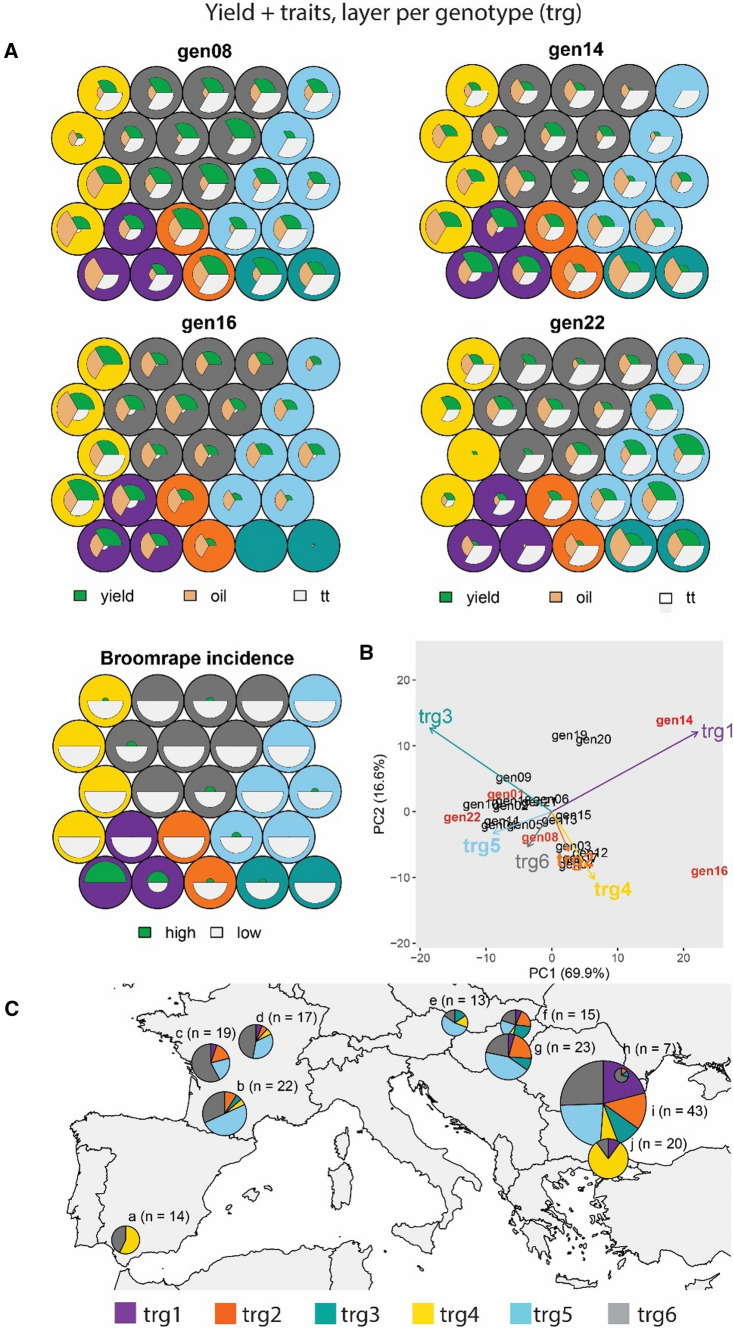
Yield + secondary traits (grain yield, thermal time to flowering, oil concentration and broomrape incidence score) one layer per genotype (tr-g). **A** Prototypes (outer circles) are coloured according to their corresponding ET. Colour codes for ETs are indicated at the bottom of the figure. For layers corresponding to gen08, gen14, gen16 and gen22, radius of the white circles is proportional to the genotype performance for each trait in trials belonging to that prototype (a larger diameter means a larger trait value, relative to the other genotypes because the yield was standardized within a trial). For the layer corresponding to broomrape incidence, prototypes are coloured according to the broomrape incidence (‘high’ or ‘low’). **B** AMMI biplot for mixed-model predicted yield of genotypes at each environment type, as identified with a self-organizing map, and **C.** Map of environment types for ‘yield + traits’. Pie sizes are proportional to the number of trials present in that geographical cluster. The ‘n’ next to each pie map indicates the number of trials in that particular geographical cluster

The input data were also organized with genotypes corresponding to individual environment × trait matrices, i.e. layers, and traits corresponding to the columns within each genotypic data matrix. We combined quantitative and qualitative traits using 23 layers (one per genotype for quantitative traits, plus one additional layer for the broomrape scores, Fig. 2). Like when we used one layer per trait, the incorporation of broomrape scores led to a separation of the trials that had a high broomrape incidence, in trg1 (Fig. 6A). trg1 mainly encompassed trials located in South Romania, Bulgaria and Turkey (pies i and j in Fig. 6C). For yield and oil concentration, gen14 and gen16 showed a strong positive interaction with trg1 trials. trg1 showed a large contrast with trg3 and trg4 trials. In trg3 trials, gen14 and gen16 had a strong negative interaction for yield. Gen22 had a positive interaction for yield with trg3. trg4 trials induced a strong positive interaction from gen16.

When comparing the relationships between traits, gen16 and gen22 had opposite yield behaviour, as visible in the radius; gen16 had very strong negative interactions for all traits in trg3 and positive interactions for yield and oil concentration in the other five ETs (Fig. 6A). In contrast, gen22 had strong positive interactions for the three traits in trg3, whereas it had negative interactions for yield and oil concentration in the other ETs. This example illustrates that the relationships between traits can help classifying environments. The relative frequency of ETs across geographical clusters indicated that trials in Spain, Turkey and South Bulgaria tend to induce different genotypic responses, due to a high frequency of trg4 trials (Fig. 6C).

### Including environmental information to characterize G × E with SOM

**Fig. 7:**
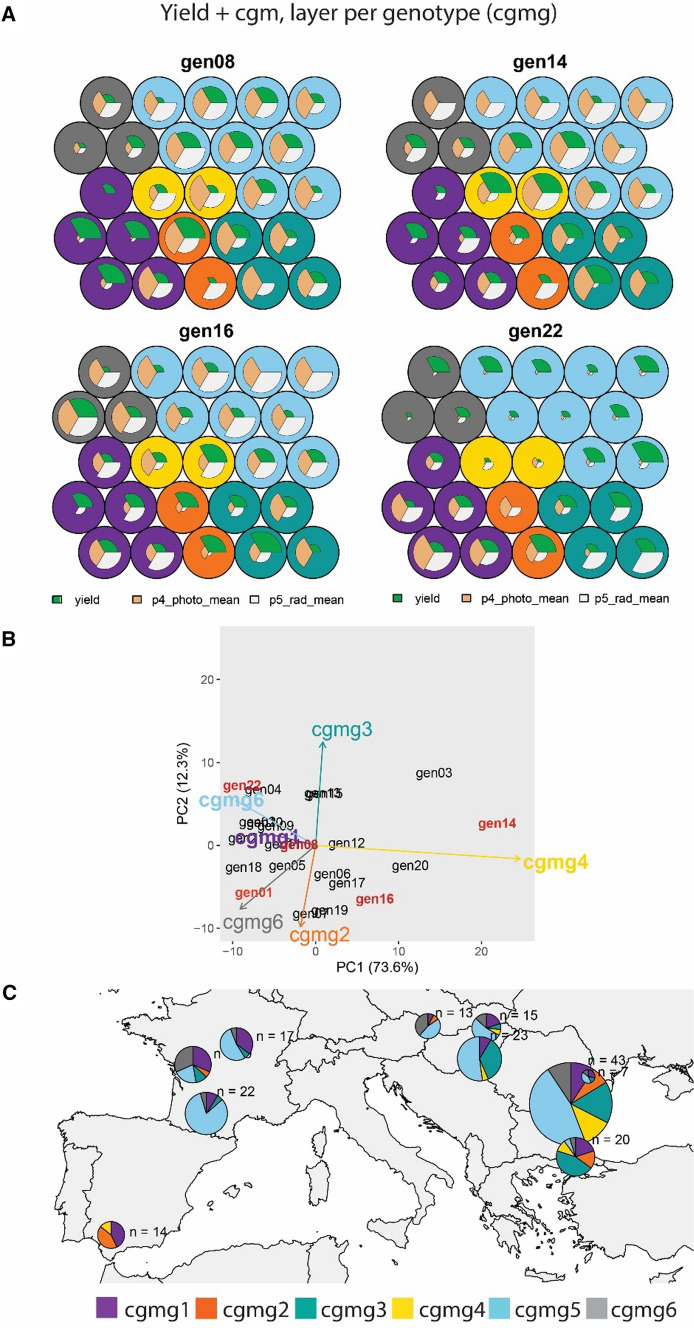
Yield + crop growth model indices, one layer per genotype (cgmg). **A** Prototypes (outer circles) are coloured according to their corresponding ET. For layers corresponding to gen8, gen14, gen16 and gen22, radius of the white circles is proportional to the genotype performance for each trait in trials belonging to that prototype (a larger diameter means a larger trait value, relative to the other genotypes because the yield was standardized within a trial). **B** AMMI biplot for mixed-model predicted yield of genotypes at each environment type, as identified with a self-organizing map, and **C.** Map of environment types for ‘yield + cgm’. Pie sizes are proportional to the number of trials present in that geographical cluster. The ‘n’ next to each pie map indicates the number of trials in that particular geographical cluster

Besides the use of several traits for environmental classification and its corresponding visualization of the genotype and environment contributions to G × E, it might be interesting to incorporate other types of information into the classification and visualization processes. For example, breeders might want to consider environmental variables to classify trials. In the example of European sunflower, the forward selection procedure in the factorial regression model indicated that the following variables were most relevant to explain G × E variation: mean photoperiod between flowering and the onset of senescence (p4_photo_mean) and mean solar radiation between the onset of senescence and maturity (p5_rad_mean). Hence, we illustrated ETs derived from SOM applied to these environmental variables, together with grain yield (Figs. 7 and S4). As for the examples using yield only and yield plus secondary traits, with data arranged as one layer per genotype, G × E was driven by ETs that occurred most often in Turkey and South of Bulgaria (pie j, Fig. 7), Romania and North of Bulgaria (pie i, Fig. 7) with Spain (pie a, Fig. 7) and with those in more Northern locations (pies b, c, d, e, f, g and h in Figs. 7 and S3).

### Adaptation zones

For all combinations of geographical clusters, trait subsets and layer arrangements, the ET classification changed across years. Hence, none of the locations can be classified with full certainty as belonging to a single ET. To identify adaptation zones that capture predictable G × E, we used ET frequencies to classify geographical clusters (locations). To do this, we used the ET frequency at each geographical cluster across years to classify them into adaptation zones. Geographical clusters that have similar ET frequencies across years will be classified as belonging to the same adaptation zone.

**Fig. 8:**
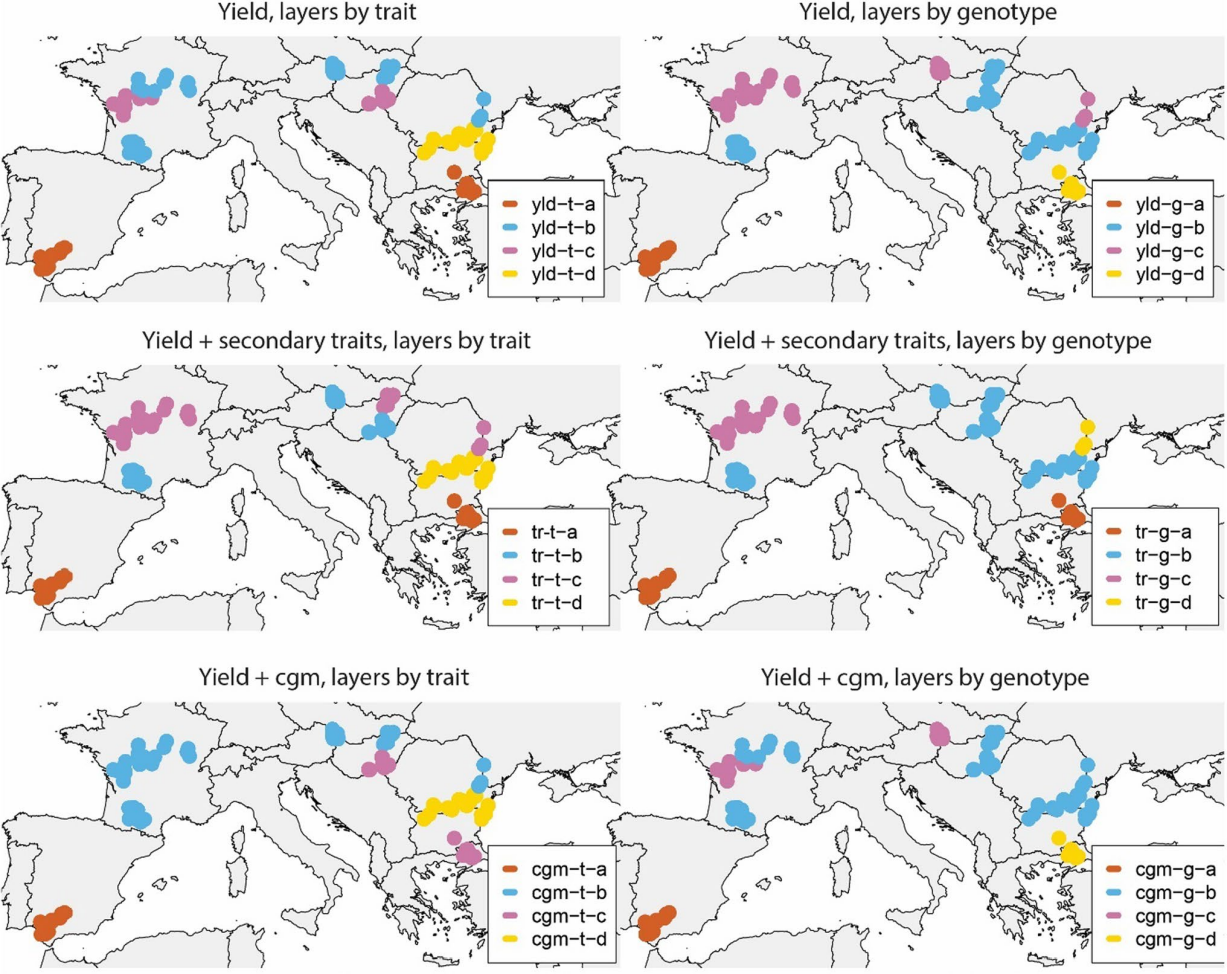
Adaptation zones based on the environment type frequencies. Environment classifications are based on a SOM with the following input data sets; yield only, yield and scores of broomrape incidence, yield and thermal time to flowering; and yield, plus Sunflo-calculated covariables. The analysis was done for data arranged as one layer per trait (left column) and as one layer per genotype, with genotypes weighted according to their contribution to G × E (right column)

**Fig. 9:**
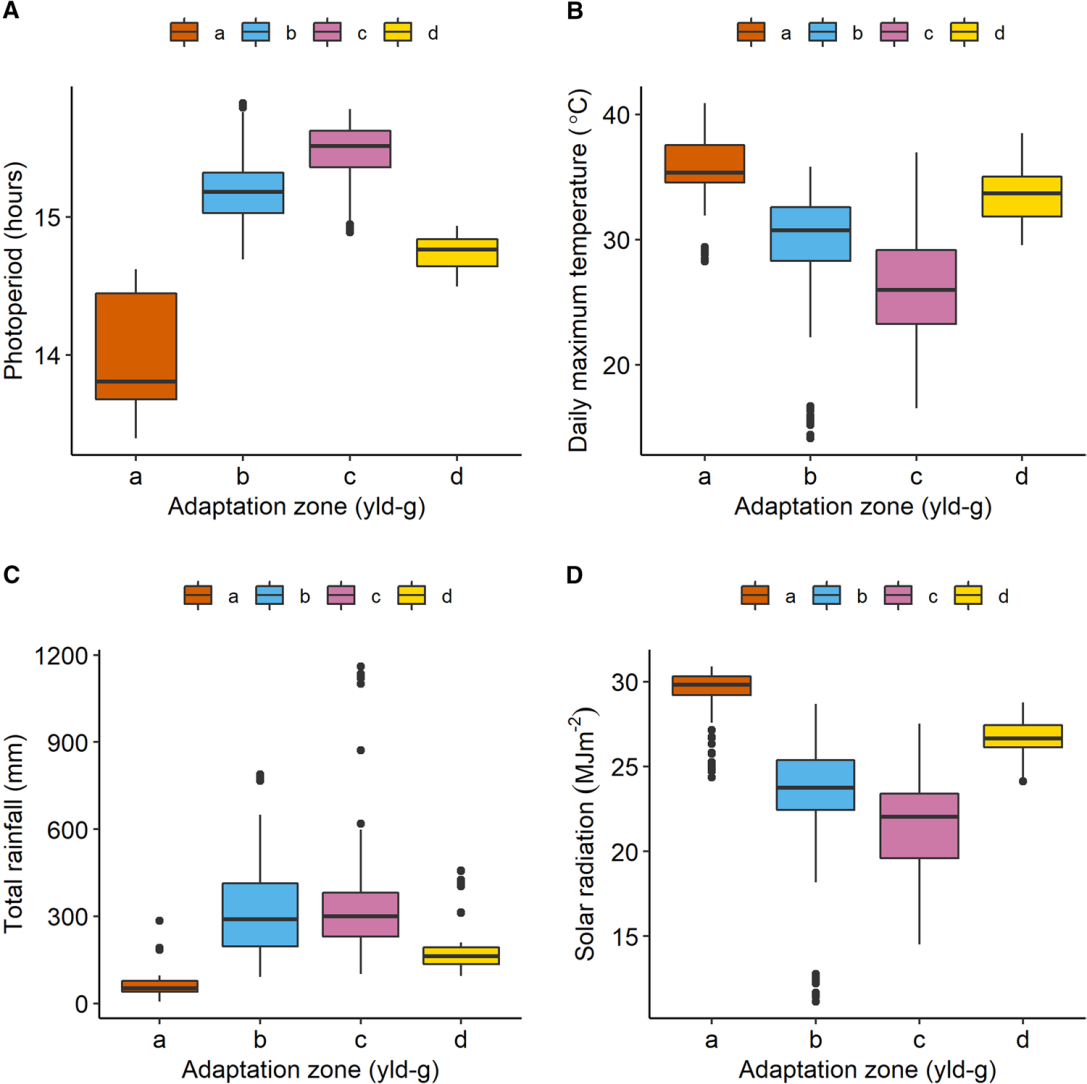
Environmental conditions at each of the adaptation zones identified with the SOM applied to the yield data with one layer per genotype (yld-g, Fig. 7, 193 trials). **A** Photoperiod between emergence and floral initiation, **B** Daily maximum temperature between the onset of senescence and maturity, **C** Rainfall between emergence and maturity. **D** Mean solar radiation between flowering and onset of senescence

Resulting adaptation zones were comparable across different input data combinations of trait subsets and layer arrangements (Table 3); trials in Spain, Turkey and South Bulgaria were most consistently classified as belonging to different adaptation zones (Fig. 8), as already observed from the ET frequency description in “Section Including environmental information to characterize G × E with SOM”. Trials in Spain (adaptation zone ‘yld-g-a’) corresponded to locations that had a shorter photoperiod (14 h), higher daily maximum temperatures (35.7 °C), lower total rainfall (79.6 mm) and higher solar radiation (29.2 MJm^−2^, Fig. 9). Trials in Turkey and South of Bulgaria (adaptation zone ‘yld-g-d’) had shorter photoperiod (14.7 h), higher temperatures (33.6 °C), reduced rainfall (199 mm) and higher solar radiation (26.8 MJm^−2^) than that in more northern locations, but their values were different from those in Spain (milder temperatures and more rainfall, Fig. 9). G × E in the remaining trials was mostly driven by an adaptation zone in France and another in South Romania. Locations in Austria and Hungary (pies e, f and g, Figs. 3, 4, 6, 7) were harder to classify due to their more variable ET assignments across trait subsets and layer arrangements. Locations in adaptation zones ‘yld-g-b’ and ‘yld-g-c’ had longer photoperiods, lower maximum temperatures, more rainfall and lower solar radiation than those in ‘yld-g-a’ and ‘yld-g-d’.

**Table 3:** Balanced accuracy for adaptation zones as obtained from different applications of SOMs in relation to a reference assignment that was provided by the yld_g SOM

Adaptation zone	yld_t	sec_t	cgm_t	sec_g	cgm_g
a	0.71	0.71	1.00	0.71	1.00
b	0.47	0.67	0.37	0.94	0.91
c	0.60	0.82	0.31	0.94	0.93
d	0.43	0.43	0.43	0.45	1.00

Yld indicates that only yield information was used, sec indicates that yield plus secondary traits were used (grain yield, thermal time to flowering, oil concentration and broomrape incidence score) and cgm indicates that yield plus crop growth model variables were used. The subscript ‘_t’ indicates that data were organized as one layer per trait, whereas ‘_g’ indicates that data were organized as one layer per genotype

To study the utility of adaptation zones, we compared the direct response to selection in adaptation zones versus indirect selection in an undivided TPE. Inspection of estimated variance components representing different sources of G × E indicated that adaptation zones explained a sizeable proportion of G × E. In all cases, the G × Z (genotype-by-adaptation zone) variance described a considerable part of the G × L variance (genotype-by-adaptation-zone + genotype-by-location-within-adaptation- zone), ranging from 0.33 (yield + CGM with one layer per trait, Table 4) to 0.58 (yield with one layer per genotype, Table 5).

**Table 4:** Variance components for genotypic main effects and various types of G × E interactions as defined by fitting a genotype (G) × location (L) × year (Y) model with locations nested within adaptation zones (Z)

Source	Yield (yld-t)		Yield + Traits (tr-t)		Yield + CGM (cgm-t)	
	Component	SE	Component	SE	Component	SE
G	11,440.1	4239.3	11,645.9	4306.6	10,873.9	4039.6
GZ	5499.5	1408.0	5540.8	1422.6	4616.7	1374.5
GY	1180.7	672.6	1210.5	671.6	725.1	721.5
GZY	3071.9	1005.6	3089.1	1006.6	4227.4	1141.7
GL(Z)	7663.3	2143.6	7854.8	2178.5	9038.1	2148.2
GL(Z)Y	30,007.1	2355.6	30,129.8	2387.6	28,867.9	2316.0
CR/DR	0.74		0.74		0.75	

Zones were based on SOMs applied to yield, yield plus secondary traits (thermal time to flowering, oil concentration and broomrape scores) and yield plus crop growth model variables (mean photoperiod between and, and mean solar radiation between and). For the SOMs, information was structured *as one layer per trait*. Environmental zones were obtained by clustering on the frequencies of environment types per geographical group. Last row gives ratio of correlated response to selection in undivided set of trials versus direct response to selection in adaptation zones

**Table 5:** Variance components and standard errors (SE) for genotypic main effects and various types of GxE interactions as defined by fitting a genotype (G) x location (L) x year (Y) model with locations nested within adaptation zones (Z)

	Yield (yld-g)		Yield + Traits (tr-g)		Yield + CGM (cgm-g)	
	Component	SE	Component	SE	Component	SE
G	9603	4132	11,840	4485	9600	4101
GZ	9595	2328	5746	1587	9044	2252
GY	1964	706	1728	715	2082	626
GZY	393	785	738	822	0	0
GL(Z)	6803	2157	8436	2173	7289	2176
GL(Z)Y	32,629	2394	31,784	2366	32,780	2375
CR/DR	0.61		0.76		0.62	

Zones were based on SOMs applied to yield, yield plus secondary traits (thermal time to flowering, oil concentration and broomrape scores) and yield plus crop growth model variables (mean photoperiod between flowering and onset of senescence, and mean solar radiation between onset of senescence and maturity). For the SOMs, information was structured *as one layer per genotype*. Environmental zones were obtained by clustering on the frequencies of environment types per geographical group. Last row gives ratio of correlated response to selection in undivided set of trials versus direct response to selection in adaptation zones

For all trait subset and layer arrangements, the CR/DR ratio was lower than 1, supporting the idea that the identified adaptation zones capture a sizeable proportion of G × E and for that reason it would pay off to select for specific adaptation. This result was also observed for the extended data set of 348 trials that only contained yield information (Figure S1). When analysing the 348 trials, G × E was driven by the contrast of locations in Spain, Turkey, South of Bulgaria, and the region around Rostov (geographical clusters a, i, j and p, Figure S1) and the more northern locations.

Across layer arrangements, the CR/DR ratio was more often smaller for the adaptation zones resulting from the data arranged in one layer per genotype, indicating that weighting genotypes by their contribution to G × E is beneficial to the identification of geographical zones with differential genotype discrimination. This might be an advantage compared to using one layer per trait, in which all genotypes receive the same weight.

Another way of evaluating the utility of selecting for specific adaptation to zones is by comparing prediction accuracies for models with and without environment classifications. We used an unstructured variance–covariance structure on adaptation zones, which allows borrowing information between adaptation zones. The adaptation zones that we identified led to a larger prediction accuracy than a genotypic main effects model without adaptation zones (Table 6), showing that the SOM combined with the post-processing of ETs was successful in classifying locations into adaptation zones that produce predictable G × E (Fig. 8). The advantage of the multi-environment model with adaptation zones was variable across different zone constructions, but prediction accuracy was for all adaptation zone models equal or larger than that for a genotypic main effects model without zones (Table 6).

**Table 6:** Mean prediction accuracy and standard error for trials in adaptation zones obtained from different applications of SOMs

Set	Layers per trait		
	Class	Accuracy	SE
Yield	yld-t-a	0.48	0.09
	yld-t-b	0.53	0.08
	yld-t-c	0.46	0.10
	yld-t-d	0.42	0.07
Yield+	tr-t-a	0.48	0.09
Secondary traits	tr-t-b	0.51	0.09
	tr-t-c	0.50	0.08
	tr-t-d	0.42	0.07
Yield+	cgm-t-a	0.46	0.08
CGM	cgm-t-b	0.52	0.08
	cgm-t-c	0.45	0.09
	cgm-t-d	0.42	0.07

Set	Layers per genotype		
	Class	Accuracy	SE
Yield	yld-g-a	0.45	0.08
	yld-g-b	0.45	0.08
	yld-g-c	0.52	0.08
	yld-g-d	0.61	0.08
Yield+	tr-g-a	0.48	0.09
Secondary traits	tr-g-b	0.45	0.09
	tr-g-c	0.54	0.07
	tr-g-d	0.63	0.07
Yield+	cgm-g-a	0.45	0.08
CGM	cgm-g-b	0.45	0.08
	cgm-g-c	0.51	0.09
	cgm-g-d	0.61	0.08

Prediction accuracies were calculated in a cross-validation scheme, leaving one year out. Benchmark prediction accuracy of the genotypic main effects model, without adaptation zones, was *0.42* ± *0.09*. Adaptation zone labels (a,b,c,d) are not necessarily consistent across input data sets

## Discussion

### The relative importance of G × E and evaluation of adaptation zones

**Fig. 10:**
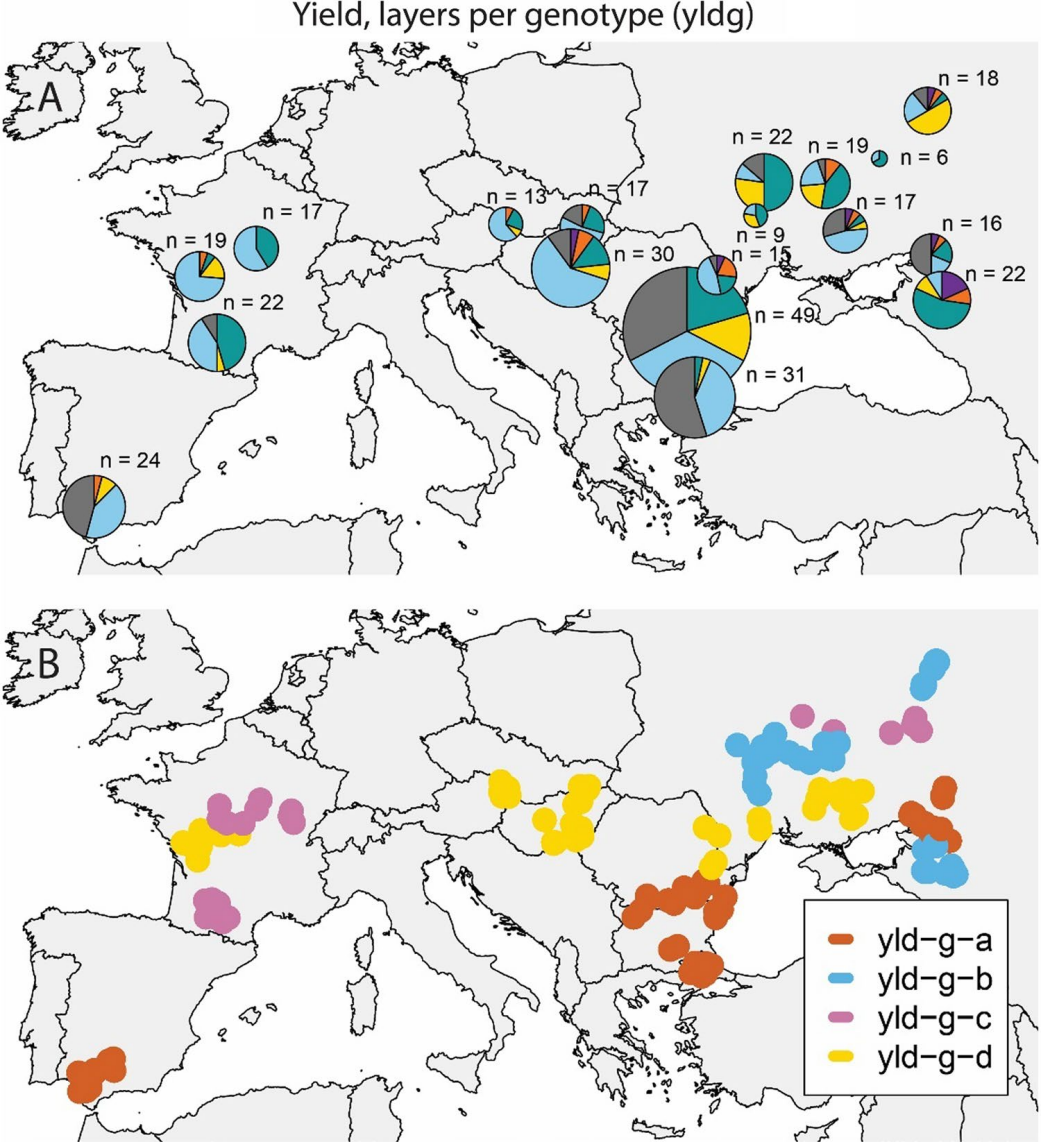
**A**. Environment types. The ‘n’ next to each pie map indicates the number of trials in that particular geographical cluster. **B**. Adaptation zones based on the environment-type frequencies for the 348 trials with yield information. Environment classifications are based on a SOM with yield only as input. The analysis was done for data arranged as one layer per genotype, with genotypes weighted according to their contribution to G × E. The CR/DR (correlated response to direct response) ratio considering the four adaptation zones was 0.63

The data set that we analysed encompassed a large range of genotypic responses, represented by hybrids of different maturity classes and adaptation patterns, and by a large range of environments that spanned a major part of the sunflower growing region of Europe (Figs. 8 and 10). Hence, although the ratio of G × E to G was a lot larger than that reported in other sunflower data sets (de la Vega and Chapman 2010), it can be considered as an upper bound for the full span of the G × E that can be expressed across the European TPE for sunflower.

When comparing the relative sizes of the variance components, Atlin et al. (2000) described that when the ratio of G × Z to G × L is large, and G × L compared to G is also large, the genetic gain strongly benefits from selecting for specific adaptation. In the example that we analysed, both the G × Z to G × L and the G × L to G ratios were very large, leading to a very low CR/DR ratio and supporting the idea of subdividing the TPE into sub-regions. The differential cultivar adaptation to more Southern regions, as we identified it, coincides to a large extent with what is currently known by sunflower breeders and growers (Velasco et al. 2015), supporting the idea that recommendation of specifically adapted genotypes is crucial for improving sunflower yields.

### Data types and data preparation

In this paper, we illustrated the use of SOMs to classify environments. We compared the resulting ETs after including a single quantitative trait (grain yield), a combination of quantitative traits (grain yield, oil concentration and, thermal time to flowering) and environment scores (grain yield and scores for broomrape pressure), and a combination of grain yield with environmental covariables calculated with the crop growth model Sunflo. These diverse data combinations illustrate the versatility of SOMs to represent and organize information of very different kinds.

One important aspect that needs to be considered when using SOMs is data preparation. We imputed and scaled the phenotypic traits per environment, i.e. per location-year combination. In that way, we remove environmental main effects and concentrate on the genotypic differences within environments. In our examples, we structured the input data for SOM as one layer per trait and as one layer per genotype. We assigned larger weights to the target trait (yield) and lower weights to the other traits. However, the SOM methodology is flexible, and layer weights can be changed as desired by the user, depending on which layer of information is of largest interest. In the current example, we assigned a larger weight to the target trait and a lower weight to other traits (a weight of 3 to yield, 2 to oil concentration, 1 to thermal time to flowering and 1 to the broomrape scores). Adaptation zones based on such weights would aim at increasing more the response to selection for grain yield than for the other traits, and simultaneously enable breeders to describe the genotypic response across environment types for multiple traits simultaneously. We evaluated several weight combinations and used the predictive ability and correlated response to selection as a criterion to define the final set of weights.

The use of data arranged as one layer per genotype is consistent with the idea that each genotype is a reference to evaluate the environment quality, which is a general approach appropriate for situations when the environmental drivers of G × E are not well identified (Fox and Rosielle 1982; Cooper and Fox 1996; Brancourt-Hulmel et al. 2000; Mathews et al. 2011). When using one layer per genotype, we decided to use the opportunity to evaluate whether assigning more weight to genotypes that are more reactive to the environment would facilitate the identification of adaptation zones to select for specific adaptation. Selecting for specific regions favours the response to selection within regions, compared to selection for broad adaptation.

In both layer arrangements (layers per trait and layers per genotype), applying SOMs to yield without any additional traits provided a meaningful environment classification. Hence, at least in the example of the European sunflower trials, there was no big gain in improving the environment classification by including additional information. However, adding more traits or environmental information does help in gaining insight about G × E for multiple traits simultaneously. Furthermore, because we use a predictive approach, predictions for yield in years that were not considered in the training set may benefit from additional information, for example, using a multi-trait prediction approach (Velazco et al. 2019). We also created SOMs based on environmental indices generated by the Sunflo crop growth model. The resulting environment classification was very similar to that obtained with yield information, showing that the use of environmental indices calculated with a crop growth model is suitable to classify environments for which no trial information is available. In our example, we didn’t have Sunflo genotype-specific parameters, other than those related to phenology. The other Sunflo parameters were constant across genotypes. Therefore, Sunflo was mainly used to define genotype-specific development periods in which environmental summaries were calculated. This implies an under-utilization of the crop growth model potential. The use of more genotype-specific Sunflo parameters could be explored in further research to explore the full crop growth model potential to explain and predict cultivar adaptation.

### Predicting responses across multiple environments

In this paper, besides the evaluation of adaptation zones via the classical approach proposed by Atlin et al. (2000), we also calculated prediction accuracy obtained with an unstructured variance–covariance matrix at the level of the adaptation zones, estimated with a mixed model. This approach has the advantage of automatically taking into account that adaptation zones might be correlated, maximizing the probability of borrowing information across adaptation zones (Piepho and Möhring 2005; Buntaran et al. 2019). It also takes into account that adaptation zones potentially can be of very different sizes, an issue that is ignored in the Atlin et al. (2000) approach.

When the focus is on selecting for specific locations (i.e. specific geographical regions), it is possible to benefit from the environment-type approach, by predicting genotype performance for each trial location by a weighted combination of the performance at each environment type and the relative frequency with which these environment types occur at each location. Such frequencies could be estimated, for example, using crop growth models that use long-term weather data as input (Chenu et al. 2013). This approach would also be useful, for example, in climate change scenarios, in which the relative frequency of hot and dry environments is expected to increase in the future (Ababaei and Chenu 2020).

We focused on the use of phenotypic traits for classifying trials into adaptation zones. At the genetic level, implementing a selection strategy that emphasizes specific adaptation will lead to changes in allele frequencies for QTLs conferring such adaptation, as shown in soybean (Kurasch et al. 2017) and maize (Millet et al. 2016). When explicit genotypic information is available in the form of molecular markers, we can improve our SOM approach to adaptation zones by augmenting our phenotypic trait information with molecular markers representing QTLs related to repeatable GxE, and thereby strengthening the genetic signal supporting the environment classification. Such an approach would enable a more detailed characterization of the adaptation landscape.

## Conclusions

- The SOM was useful to classify trials into environment types that make an important contribution to G × E.
- SOM was useful to visualize G × E in a two-dimensional grid that describes the adaptive responses of each genotype.
- The sunflower data that we analysed contains substantial G × E. Most of it is driven by G × L × Y (non-repeatable G × E), but there is also an important contribution of G × L (predictable G × E), opening the possibility to classify locations into adaptation zones.
- We used the distribution of ETs per location to classify locations into adaptation zones that were effective in capturing G × L.
- Arranging data in one layer per genotype and weighting genotype layers by their contribution to G × E led to adaptation zones that were more effective in capturing G × E than when data were arranged as one layer per trait (hence, with all genotypes weighted equally).
- The resulting ETs and adaptation zones slightly varied depending on the input information. However, they coincided to a large extent in pointing out trials in Spain, Turkey and South Bulgaria as inducing a different genotypic response.

## Supplementary Information

Below is the link to the electronic supplementary material.

Supplementary file1 (DOCX 1417 kb)

## Data Availability

The data that support the findings of this study are available from Corteva Agriscience. Restrictions apply to the availability of these data, which were confidentially used under license for this study.
