# Supplementary material for: Identification of environment types and adaptation zones with self-organizing maps; applications to sunflower multi-environment data in Europe
Source: Theor Appl Genet. 2022 May 7;135(6):2059–82. doi: 10.1007/s00122-022-04098-9 (PMC9205840; doi:10.1007/s00122-022-04098-9)
Supplement: Supplementary file 1 — Supplementary file1 (DOCX 1417 kb) [file 122_2022_4098_MOESM1_ESM.docx]

**Supplementary Figures**

**
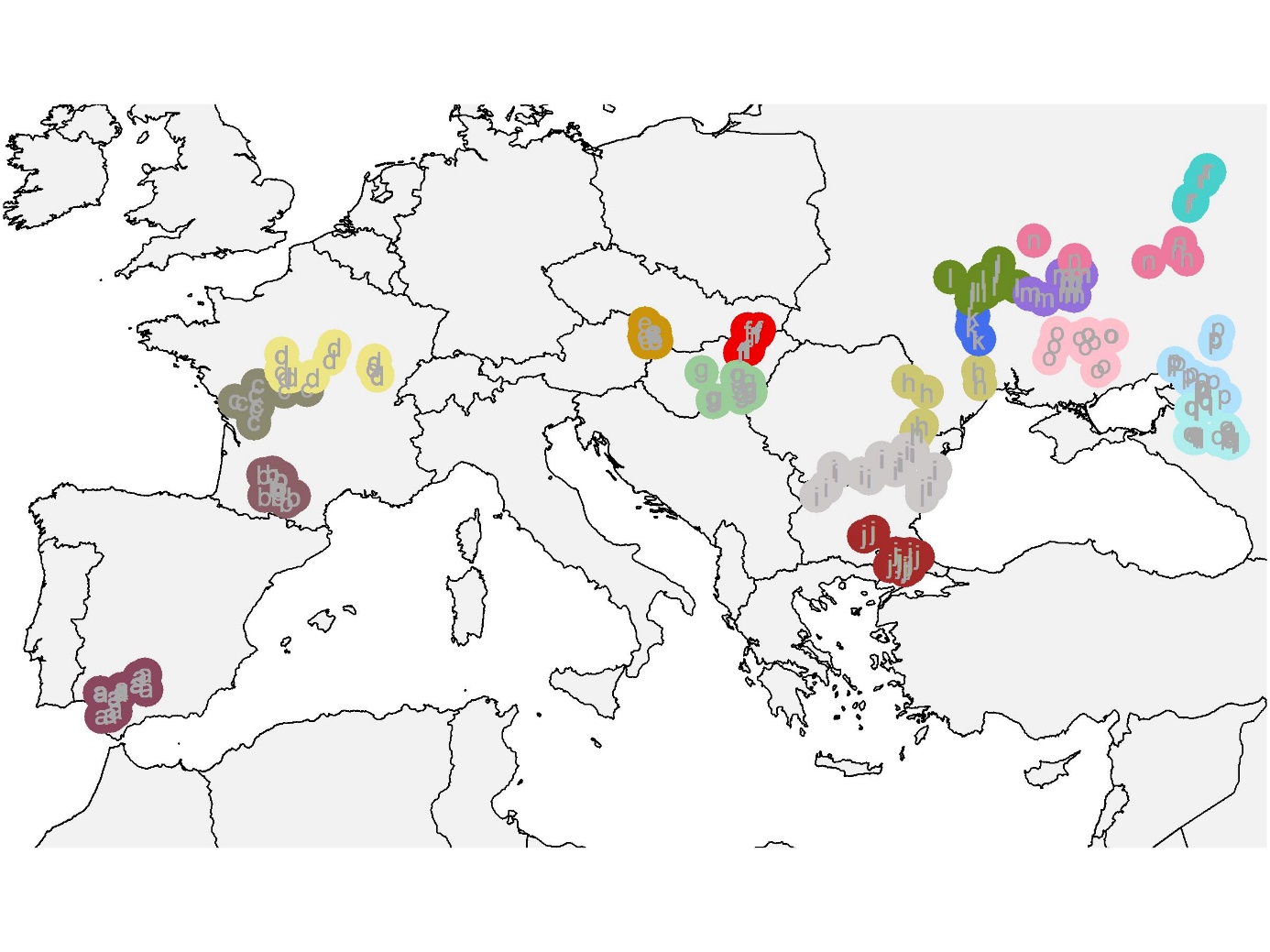
**

Figure S1. Geographical clusters of sunflower trials. Trials belonging to the same cluster are indicated with the same colour and letter. Clusters were formed using latitude and longitude information as input to a SOM. Clusters ‘a’ to ‘h’ had yield and oil concentration phenotypes, whereas clusters ‘i’ to ‘r’ had only yield phenotypes.


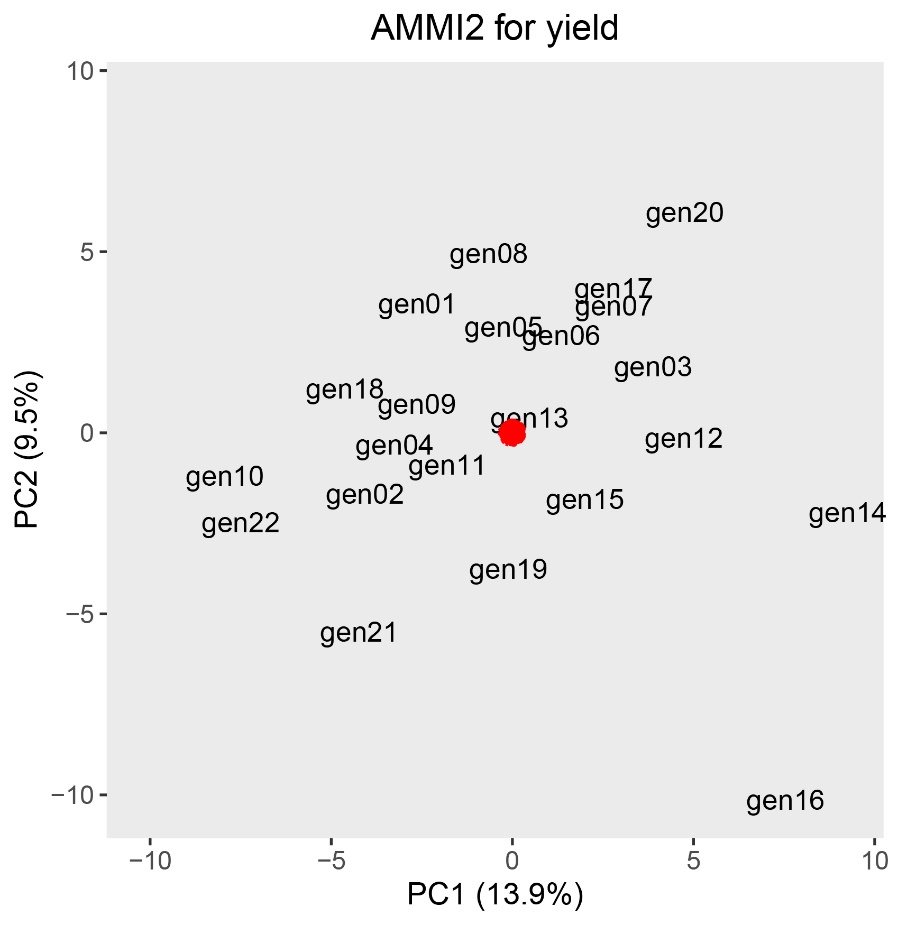


Figure S2. AMMI biplot for yield of 22 genotypes in 193 environments. AMMI genotype scores were used to determine weights in SOMs.


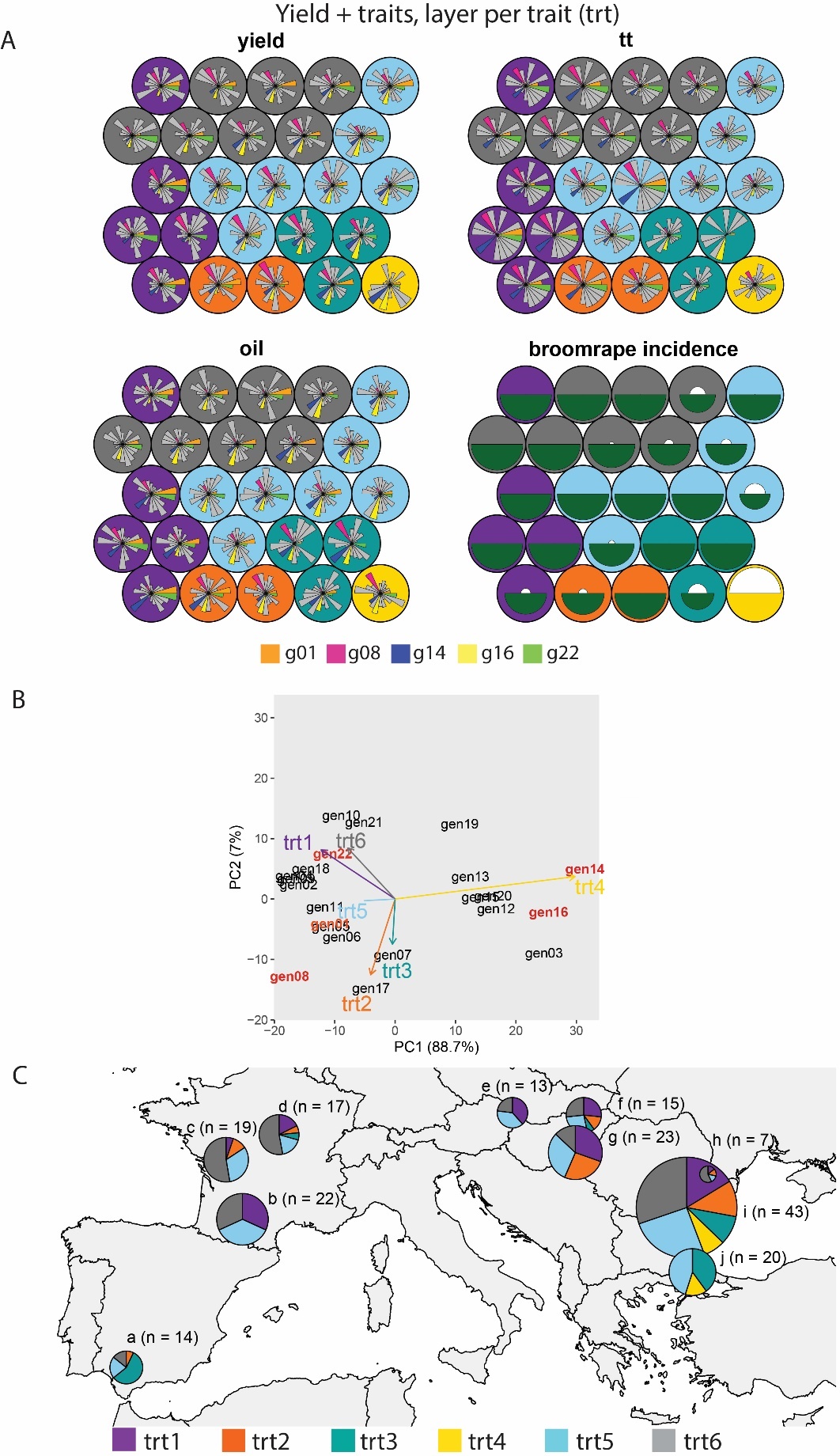


Figure S3. **Yield + traits, layer per trait (trt).** **A.** Prototypes (outer circles) are coloured according to their corresponding ET. Genotypes are indicated in sequential order from g01 to g22. Genotypes g01, g08, g14, g16 and g22 are highlighted in colour. Radius of each genotype is proportional to the genotype performance in trials belonging to that prototype (a larger diameter means a larger yield, relative to the other genotypes because the yield was standardized within a trial). **B.** AMMI biplot for yield predictions of genotypes with respect to each environment type, as identified with a self-organizing map and **C.** Map of environment types Pie sizes are proportional to the number of trials present in that geographical cluster.


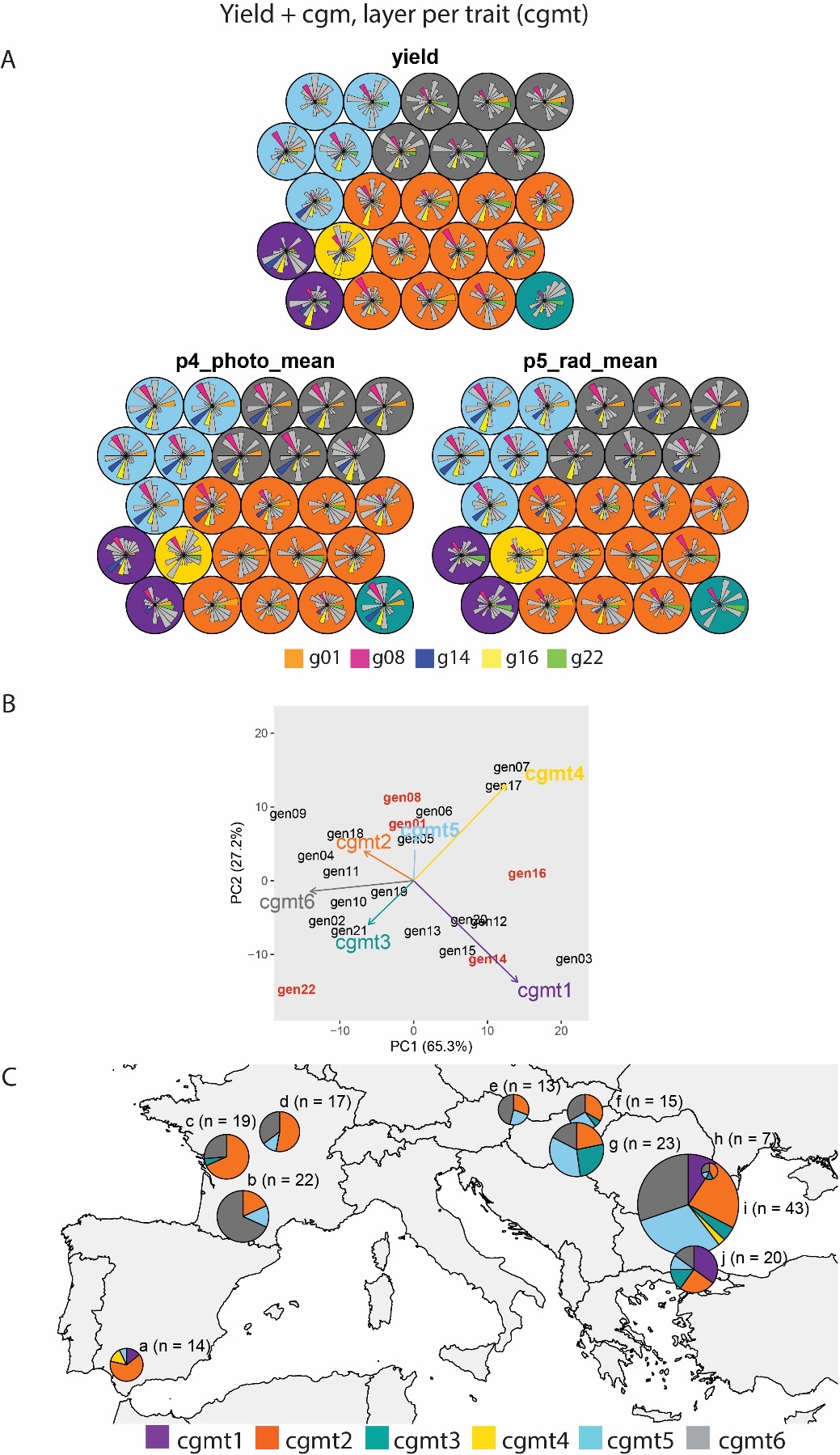


Figure S4. Yield + crop growth model indices (p4_photo_mean and p5_rad_mean), one layer per trait (cgmt). **A.** Prototypes (outer circles) are coloured according to their corresponding ET. Genotypes are indicated in sequential order from g01 to g22. Genotypes g01, g08, g14, g16 and g22 are highlighted in colour. Radius of each genotype is proportional to the genotype performance in trials belonging to that prototype (a larger diameter means a larger yield, relative to the other genotypes because the yield was standardized within a trial). **B.** AMMI biplot for yield predictions of genotypes at each environment type as identified with a self-organizing map and **C.** Map of environment types. Pie sizes are proportional to the number of trials present in that geographical cluster.

**Supplementary tables**

Table S1. Parameter values that were used to run the Sunflo crop growth model at each of the 348 environments.

| **Symbol** | **Label** | **Description** | **Default value** | **Unit** | **Used value** | **Explanation used value** |
| --- | --- | --- | --- | --- | --- | --- |
| TDE1 | Thermal Time Vegetative | Temperature sum to floral initiation | 482 | ⁰Cd | TDE1=TDM0/2 | Similar value to the one reported in (Debaeke et al. 2010; Debaeke and Izquierdo 2021) for several cultivars. |
| TDF1 | Thermal Time Flowering | Temperature sum from emergence to the beginning of flowering | 836 | ⁰Cd | TDE1=TDM0/1.3 | To keep proportionality of TDM0/TDF1 as in default parameters |
| TDM0 | Thermal Time Senescence | Temperature sum from emergence to the beginning of grain filling | 1083 | ⁰Cd | observed TT for each G-E combination |  |
| TDM3 | Thermal Time Maturity | Temperature sum from emergence to seed physiological maturity | 1673 | ⁰Cd | TDF1 + 750 | Agree with Rondanini et al., (2007), who show that the grain filling duration is around 750⁰Cd |
| TLN | Potential Leaf Number | Potential number of leaves at flowering | 29 | leaf | default | Agree with Gimenez et al., (1994) |
| LLH | Potential Leaf Profile | Potential rank of the plant largest leaf at flowering | 17 | leaf | default | Agree with Gimenez et al., (1994) |
| LLS | Potential Leaf Size | Potential area of the plant largest leaf at flowering | 448 | cm^-2^ | 1000 | largest value reported by Bange et al., (2000) |
| k | Extinction Coefficient | Light extinction coefficient during vegetative growth | 0.88 | - | default |  |
| LE | Water Response Expansion | Threshold for leaf expansion response to water stress | -4.42 | - | default |  |
| TR | Water Response Conductance | Threshold for stomatal conductance response to water stress | -9.3 | - | default |  |
| HI | Potential Harvest Index | Potential harvest index | 0.4 | - | default |  |
| OC | Potential Oil Content | Potential seed oil content | 55.4 | % dry | default |  |

Table S2. Environmental indices to characterize the 348 environments representing the European sunflower TPE, calculated at the genotype level for each of the four growing periods considered in the analysis; from crop emergence to floral initiation (p2), from floral initiation to flowering (p3), from flowering to onset of senescence (p4) and from onset of senescence to maturity (p5).

| **Class** | **Name** | **Description** |
| --- | --- | --- |
| Temperature | tmin | Minimum daily temperature |
|  | tmax | Maximum daily temperature |
|  | tmean | Mean daily temperature |
| Rainfall | rain_sum | Rainfall sum |
| Solar radiation | rad_mean | Mean photoperiod |
| Sunflo growth output | ETPET | Reference evapotranspiration |
|  | FHRUE | Photosynthesis response to water stress |
|  | FHTR | Transpiration response to water stress |
|  | FNRUE | Photosynthesis response to nitrogen stress |
|  | FTRUE | Photosynthesis response to thermal stress |
|  | FTSW | Fraction of transpirable soil water |
|  | GR | Global incident radiation |
|  | GY | Grain yield |
|  | LAI | Leaf area index |
|  | NAB | Absorbed nitrogen |
|  | NNI | Nitrogen nutrition index |
|  | OC | Grain oil content |
|  | RIE | Radiation interception efficiency |
|  | RR | Rainfall |
|  | RUE | Radiation use efficiency |
|  | TM | Mean air temperature |
|  | TN | Minimum air temperature |
|  | TX | Maximum air temperature |
| Sunflo total biomass | TDM | Crop aerial dry biomass |
| Phenology | TDE1 | Temperature sum to floral initiation |
|  | TDF1 | Temperature sum from emergence to the beginning of flowering |
|  | TDM0 | Temperature sum from emergence to the beginning of grain filling |
|  | TDM3 | Temperature sum from emergence to seed physiological maturity |
|  | TTA2 | Temperature sum from emergence |
|  | flowering (in tt) | Thermal time to R1 |
